# Behavior and Chemical Ecology in Phoridae (Diptera): A Systematic Review of Parasitoid-Host Interactions

**DOI:** 10.1007/s13744-026-01431-5

**Published:** 2026-08-31

**Authors:** Catarina Silva Correia, Milena Gama Oliveira, Airton Torres Carvalho, Artur Campos Dália Maia

**Affiliations:** 1 Programa de Pós-Graduação em Biologia Animal, Centro de Biociências, Universidade Federal de Pernambuco, Recife, Pernambuco Brazil; 2 Programa de Pós-Graduação em Ecologia e Conservação da Biodiversidade, Universidade Estadual de Santa Cruz, Ilhéus, Bahia Brazil; 3 Centro de Ciências Biológicas e da Saúde, Departamento de Biociências, Universidade Federal Rural do Semi-Árido, Mossoró, Rio Grande Do Norte Brazil

**Keywords:** Ecological network, Parasitism, Phorid flies, Semiochemicals

## Abstract

Phorid flies are a highly diverse group that exhibits multiple parasitic strategies involving ants, bees, and other invertebrates. Although comprehensive reviews are limited, most studies emphasize parasitoid–host interactions, especially within *Apocephalus*, known as “decapitating flies” because their larvae develop inside ant heads. Some species, such as *Pseudohypocera kerteszi* (Enderlein, 1912), are economically important due to their negative effects on stingless bee colonies. Chemical ecology plays an important role in understanding these interactions, as compounds like cuticular hydrocarbons and mandibular secretions function as semiochemicals guiding host recognition and influencing parasite behavior. However, studies addressing chemical mechanisms remain relatively limited. This review systematically synthesizes data on parasite–host interactions in Phoridae, integrating behavioral and chemical perspectives. We also conducted an ecological network analysis at the genus level to identify functional roles and interaction patterns among phorids and their hosts. The results highlight a predominance of parasitoid interactions and a strong focus on ant-associated systems, which may reflect research bias. Overall, our findings contribute to a better understanding of the ecological strategies and community-level implications of parasitism in this diverse and behaviorally complex fly family, while also highlighting gaps in current knowledge.

**Supplementary Information:**

The online version contains supplementary material available at https://doi.org/10.1007/s13744-026-01431-5.

## Introduction

Diptera is a highly diverse and globally distributed insect order, with particularly high diversity in the Neotropical region (Disney 1994). Within this group, the family Phoridae, which is the third-largest parasitic dipteran family, stands out for its remarkable diversity of life-history strategies, including parasitoidism, kleptoparasitism, predation, and, less commonly, herbivory (Sivinski et al. 1999; Brown 2010; Li et al. 2025). Despite its ecological and economic importance, the internal classification of Phoridae remains unresolved. Traditional morphology-based classifications recognize multiple subfamilies (e.g., Sciadocerinae, Chonocephalinae, Termitoxeniinae, Metopininae, and Phorinae) (Brown et al. 2015), whereas recent molecular phylogenies have challenged the monophyly and relationships among these groups (Li et al. 2025). This taxonomic uncertainty has important implications for interpreting ecological patterns, including host use and specialization.

Phoridae are the most ecologically diverse insects and interact with a wide range of hosts, particularly social insects such as ants and bees, but also other arthropods and, more rarely, vertebrates (Villa et al. 1983; Holcomb and Carr 2011; Francati 2015; Ament and dos Santos 2017). Among these interactions, parasitoidism is the most extensively studied strategy, especially in systems involving ants, where larvae develop within the host body and can significantly affect host survival and colony dynamics (Brown and Feener Jr 1991; Feener Jr and Brown 1993; Brown 2010). In this review, we use the term “parasite–host interactions” in a broad sense to encompass parasitoidism, parasitism, predation, and kleptoparasitism, which are defined explicitly in the Methods.

Chemical ecology plays a central role in mediating these interactions, particularly in systems involving social insects, where semiochemicals regulate communication and behavior (Howard and Blomquist 1982, 2005). These chemical cues are involved in host detection, recognition, and exploitation, and may influence the degree of host specificity. In addition, behavioral strategies such as inspection flights, selective oviposition, and host handling further shape interaction outcomes (Feener et al. 1996; Mathis and Tsutsui 2016). However, most studies have examined these mechanisms in isolation, often focusing on specific taxa or interaction types.

Although previous syntheses have addressed aspects of phorid biology (Sivinski et al. 1999), an integrative perspective combining chemical ecology, behavior, and ecological structure remains lacking. This gap is further compounded by taxonomic uncertainty and uneven research effort across taxa and regions, which may bias our understanding of interaction patterns.

Here, we propose that variation in host specialization in Phoridae can be understood as an emergent property of the interaction between (i) the type and specificity of chemical cues used for host detection and (ii) the behavioral strategies employed during host location and oviposition. Systems relying on highly specific cues, such as host-specific pheromones or cuticular signatures, are expected to favor specialization, whereas those exploiting more general cues, such as disturbance signals or broadly shared chemical compounds, may facilitate generalist strategies. By integrating evidence from chemical ecology, behavioral studies, and ecological network analysis, this review aims to synthesize current knowledge and explore how these mechanisms contribute to patterns of host use and ecological diversification in Phoridae.

## Search Methodology

We adopted a methodology based on the Preferred Reporting Items for Systematic Reviews and Meta-Analyses (PRISMA 2020) guidelines (Page et al. 2021). We included peer-reviewed articles published in English or Portuguese up to 2024, and selected articles focused on Phoridae, including host–parasite relationships, defensive behaviors, chemical ecology, and biological control. Literature searches were conducted in the Scopus and Web of Science databases using the keywords “phorid” and “forídeo,” applied to titles, abstracts, and keywords. The final search was performed in December 2024.

Regarding the exclusion criteria, studies were excluded if they focused on topics not directly related to biological interactions involving Phoridae (e.g., plant or fungal associations, systematics, morphology, genetics, forensic entomology, necrophagy, or dispersal). We also excluded studies that did not explicitly address phorid–host interactions, did not identify phorids at least to the genus level, or included multiple Diptera families without clear attribution of interactions to Phoridae. The screening process was conducted in two stages. First, titles and abstracts were evaluated for relevance based on the criteria. Subsequently, full-text screening was performed to confirm eligibility. 

To ensure terminological consistency, interaction types were classified according to standard ecological definitions. Parasitoids were defined as organisms whose immature stages develop on or within a single host, ultimately leading to host death (Price 1980; Godfray 1994). Parasites exploit hosts without necessarily causing immediate mortality (Price 1980), whereas predators consume multiple prey individuals over their lifetime. Kleptoparasites were defined as species that exploit resources collected or produced by their hosts (Begon et al. 2006). These definitions were used to standardize the classification of interaction strategies across studies.

## Ecological Network

We used ecological network analysis to evaluate interactions recorded in the literature between phorid species and their hosts. A qualitative (presence–absence) bipartite network was constructed, with interactions aggregated at the genus level to minimize inconsistencies in species-level identification across studies. Species-level metrics were applied to examine the functional roles of individual taxa within the bipartite host–phorid network.

Species-level indices were computed using the specieslevel function from the bipartite package in R. We focused on two key metrics: closeness centrality, which measures how close a taxon is to all others in the network based on the shortest paths connecting them, which shows how easily a taxon can reach or affect others in the network, and the specialization index *d*′, which quantifies how selectively a taxon interacts with its partners relative to a null expectation, with higher values indicating greater ecological specialization. These metrics were calculated separately for hosts (lower level) and phorid taxa (higher level). To explore the structural roles of taxa within the network, we ranked them according to their closeness and *d*′ values and visualized the 15 most central or specialized taxa in each group.

The selection of these metrics aimed to capture complementary aspects of network structure, combining measures of connectivity (closeness) and interaction selectivity (*d*′). However, because the network is derived from published literature rather than standardized sampling, interaction records likely reflect research effort rather than ecological frequency or abundance. As a result, network position should be interpreted cautiously, and centrality may be influenced by sampling bias toward well-studied taxa. Additionally, the aggregation of interactions at the genus level may obscure species-specific patterns of specialization. Therefore, the network analysis is intended primarily as an exploratory tool to identify broad patterns of interaction structure, rather than to infer definitive ecological roles.

## Study Selection

**Fig. 1:**
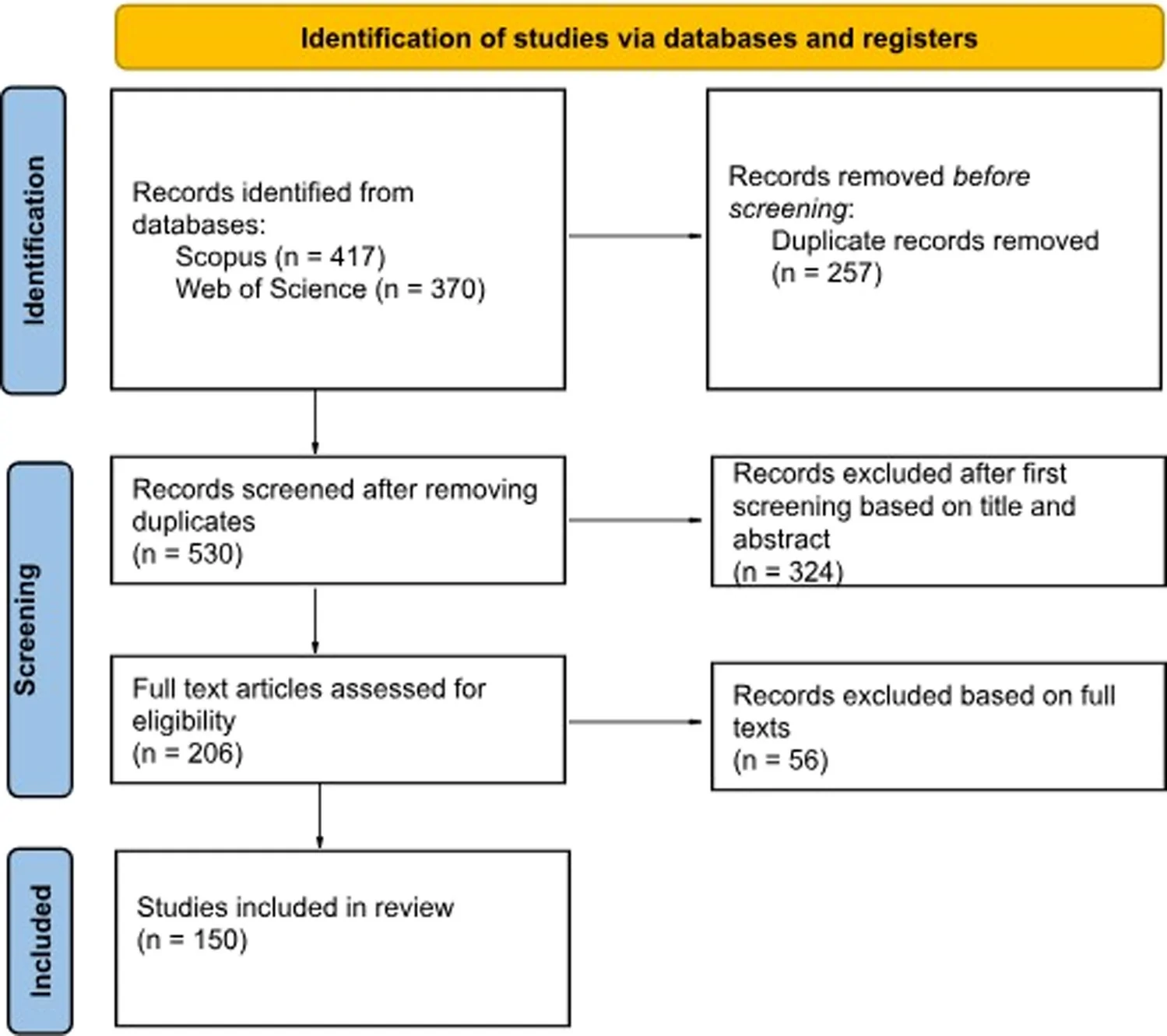
PRISMA (2020) flow diagram illustrating the literature search and study selection process, including identification, screening, eligibility, and inclusion of studies on parasite–host interactions involving Phoridae

There were 787 registered studies, of which 417 were found on Scopus and 370 on Web of Science. After removing 257 duplicates using EndNote, 530 unique records remained for screening. In the first stage, titles and abstracts were screened for relevance, resulting in the exclusion of 324 records that did not meet the inclusion criteria. As a result, 206 studies were selected for a comprehensive reading. In this last screening process, 150 articles were officially included, and 56 were excluded based on the exclusion criteria. The study selection process is summarized in the PRISMA flow diagram (Fig. 1).

### General Characteristics of the Included Studies 

Based on the recent Phoridae phylogeny by Li et al. (2025), the species reviewed belong to the Metopininae and Phorinae subfamilies. The updated classification reassigns *Termitophilomyia zimbraunsi* Disney 1994 to Phorinae, previously placed in Termitoxeniinae (Brown et al. 2015). The uncertain position of genera such as Ctenopleuriphora, which shares traits with both subfamilies, indicates a complex evolutionary history. The presence of host-associated interaction strategies across distant branches suggests that these strategies may have evolved multiple times within Phorinae, emphasizing the need for integrative phylogenetic studies to clarify evolutionary patterns and ecological diversification in this diverse fly family.

The reviewed literature reveals a strong focus on specific interactions and host groups. Parasitoidism was the overwhelmingly dominant relationship studied, featured in 91% of publications. In these interactions, phorid larvae develop within a host, ultimately leading to host death. This process typically begins with a female phorid fly rapidly ovipositing into a specific part of the host’s body, such as the head (Feener Jr and Brown 1993; Bragança et al. 2009), thorax (Brown and Feener 1991a, b; Cônsoli et al. 2001), abdomen (Core et al. 2012), or between the sclerites (Hash et al. 2017). Upon hatching, the larvae consume the host’s internal tissues, leading to host mortality (Mccabe 1998; El-Hawagry et al. 2021).

While far less common, non-parasitoid interactions were also documented. These include parasitism and kleptoparasitism. Kleptoparasites exploit resources collected or produced by their hosts (Sivinski et al. 1999). For instance, some *Megaselia* species lay eggs in the brood cells of wasps, where their larvae consume available resources within the host nest (Itino 1986).

The choice of host was also highly concentrated, with ants being the subject of over 76% of all studies. A significant but smaller portion of research focused on bees (8.0%), followed by beetles, wasps, and frogs (approx. 2.7% each). A wide variety of other host organisms, including bugs, spiders, termites, and millipedes, were documented in less than 2% of studies each, indicating that the full host range of Phoridae remains a largely unexplored field.

**Fig. 2:**
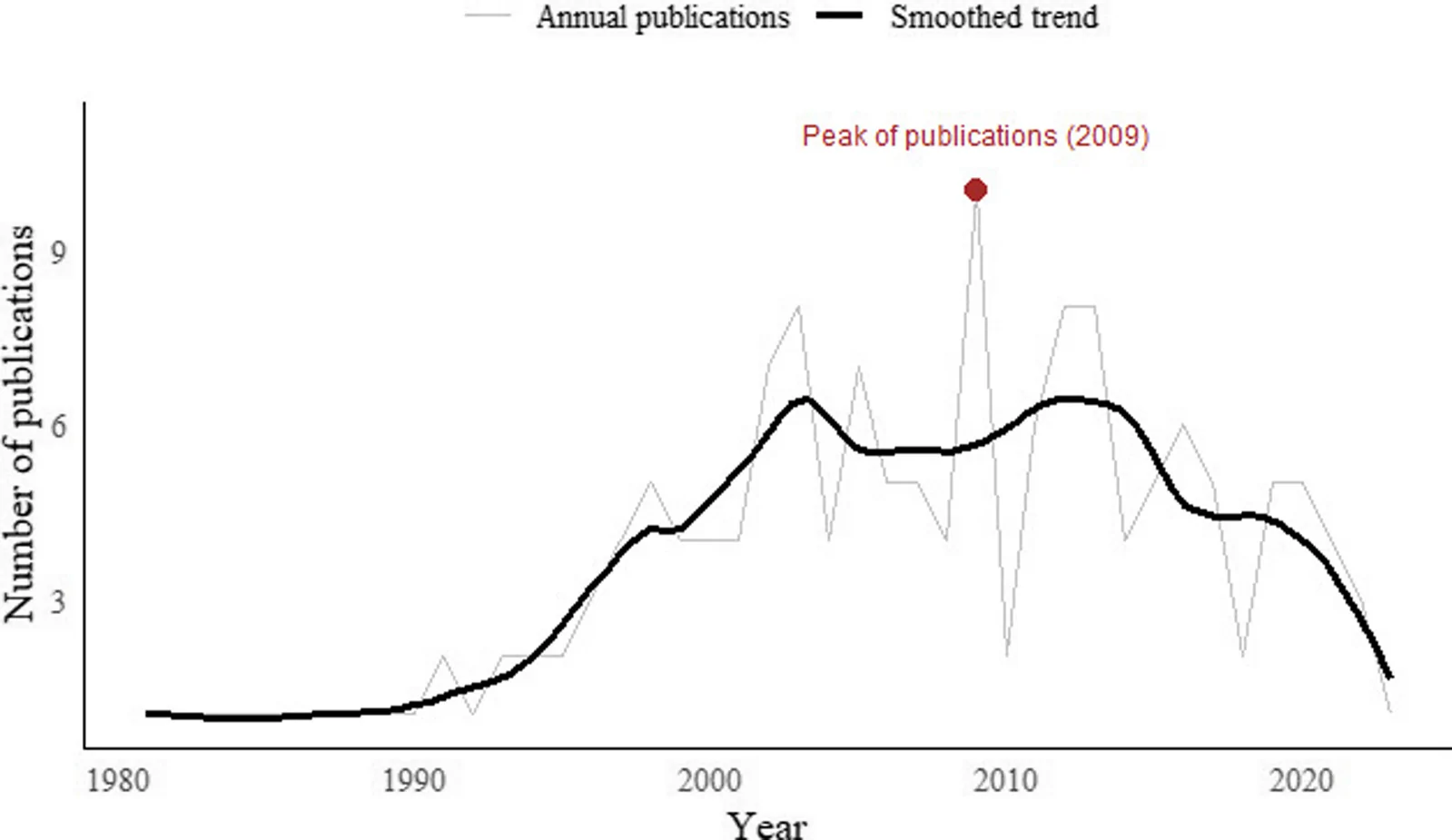
Temporal trend in the number of publications on parasite–host interactions involving Phoridae from 1981 to 2024. Gray lines represent the annual number of publications, while the black line indicates the smoothed trend. The red point highlights the peak in publication output (2009)

The studies reviewed were published between 1981 and 2024. The volume of research increased significantly in the 2000 s, peaking in 2009 before declining in recent years (Fig. 2). This body of research has been geographically concentrated in the Americas. The USA (34%) and Brazil (28%) together account for over 60% of the published work. Other significant contributions have come from Latin American countries, including Argentina (11%), Costa Rica (8%), and Mexico (4%). The remaining publications were distributed across more than 20 countries, with each contributing less than 2% of the total literature.

The parasitoid behavior of some phorid flies makes them valuable biological control agents, especially against invasive insects. This is exemplified by their use in managing the red imported fire ant (*Solenopsis invicta* Buren, 1972) in the USA, a major ecological pest that prompted extensive research into control strategies (Porter 1998). Native *Pseudacteon* species have been widely studied for their effectiveness in suppressing fire ant populations (Mottern et al. 2004; Oi et al. 2019). Likewise, *Apocephalus attophilus* Borgmeier, 1928, shows promise for controlling leafcutter ants (*Atta bisphaerica* Forel, 1908) due to its high fecundity and rapid larval development (Farder-Gomes et al. 2018).

This strong economic interest in controlling pest ants likely fueled the surge in publications observed during the 2000 s, especially in the USA. In stark contrast to the intense focus on ants, interactions involving other hosts remain significantly understudied. For example, while the association between phorid flies and bees is well recognized by beekeepers (Nogueira-Neto 1997), it has received comparatively little attention in international scientific journals. This reveals notable knowledge gaps in the field, particularly in understanding the chemical ecology that mediates the attraction of phorids to bee colonies, representing a key gap and a promising direction for future research.

## Study Genera and Species: Ecological Network 

**Fig. 3:**
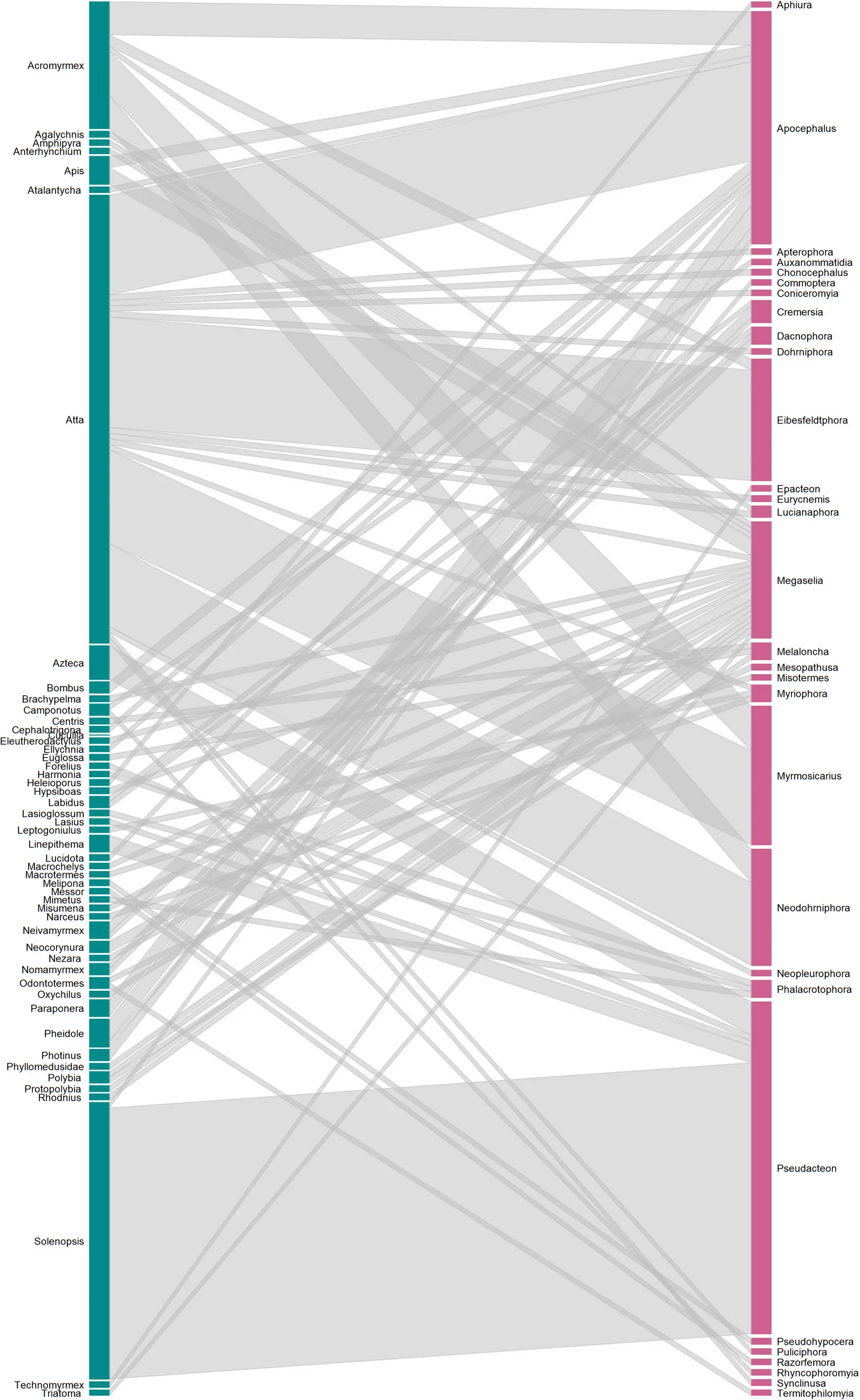
Bipartite ecological network showing interactions between Phoridae genera (right) and their host genera (left), based on records compiled from a systematic review of the literature up to 2024. Lines represent reported host–parasitoid associations

**Fig. 4:**
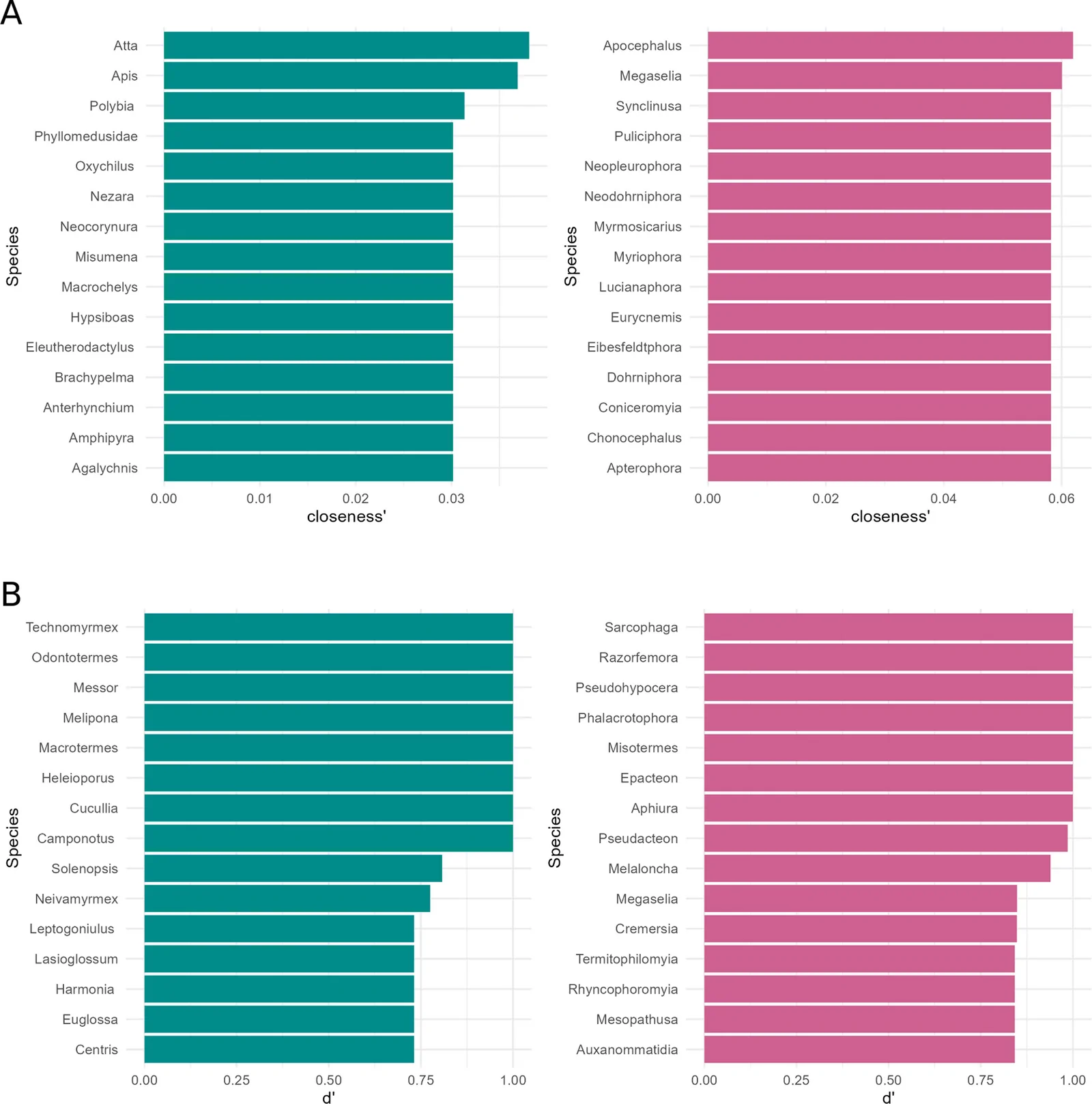
Network metrics describing interaction patterns between Phoridae parasitoids and their hosts. **A** Closeness centrality values for host genera (left) and Phoridae genera (right), indicating their relative position and connectivity within the interaction network. **B** Specialization index (*d*′) for hosts (left) and Phoridae genera (right), reflecting differences in interaction breadth and the degree of ecological specialization

The ecological network of interactions between phorid flies and their hosts is illustrated in Fig. 3. An analysis of the interaction network using closeness centrality and specialization index (*d*′) reveals the structural importance of key genera within both host and parasitoid groups (Fig. 4). Among the hosts, the genera *Atta* (leafcutter ants) and *Apis* (honey bees) exhibited the highest centrality values. Among the Phoridae, *Apocephalus* and *Megaselia* emerged as the most central, suggesting they function as critical interaction hubs that connect to a wide array of hosts. It is crucial to acknowledge that these results are likely influenced by research bias. The high centrality of certain genera may reflect their prominence in the scientific literature rather than their true ecological role alone. Additionally, because interactions were aggregated at the genus level, the network may mask species-level variation in host use, which should be considered when interpreting these patterns.

For instance, *Atta* is considered a major agricultural pest in the USA, known for its complex social organization and vast nests, which have made it a primary focus of pest management research (Zanetti et al. 2014; Della Lucia et al. 2014). This intense research focus has documented its interactions with a diverse group of phorid parasitoids, including *Apocephalus*, *Eibesfeldtphora*, *Myrmosicarius*, and *Neodohrniphora* (Bragança et al. 2009; Farder-Gomes et al. 2018; de Almeida et al. 2023). Similarly, *Apis* is extensively studied due to its immense ecological and economic importance as a pollinator for both native and cultivated plants, making it vital for sustainable agriculture (Partap 2011; Singh 2022). This research interest has solidified its central position in the network. Interestingly, the two most central phorid genera, *Apocephalus* and *Megaselia*, appear to achieve their hub status through different ecological strategies. The central host *Apis* is associated exclusively with these two phorid genera, suggesting a relatively tightly connected core within the network.

*Apocephalus* demonstrates a relatively focused host range, primarily targeting social insects like ants and bees, as well as beetles (Feener Jr 1981; Brown 1994; Core et al. 2012). In contrast, *Megaselia* is a true generalist with an exceptionally broad host range that spans multiple phyla. It has been recorded attacking not only a wide variety of insects (ants, bees, wasps, bugs) but also other arthropods (spiders) and even other animal groups entirely, such as snails, frogs, and turtles (Villa et al. 1983; Holcomb and Carr 2011; Morse 2013; Brown and Vendetti 2020). This suggests that while both genera are network hubs, *Megaselia* acts as a key link connecting disparate ecological guilds.

The specialization index (*d*′) revealed a strong pattern of host specificity, with several host genera, including *Technomyrmex*, *Messor*, *Macrotermes*, *Cucullia*, *Heleioporus*, *Odontotermes*, and *Melipona*, exhibiting the maximum specialization value (*d*′ = 1). This indicates interaction with only a single phorid genus. Correspondingly, most of their associated phorid taxa, such as *Razorfemora*, *Pseudohypocera*, *Misotermes*, and *Epacteon*, also showed maximum specialization, suggesting tight and potentially co-evolved relationships.

These specialist interactions encompass multiple ecological strategies, including parasitoidism, parasitism, predation, and kleptoparasitism. Examples include parasitoid associations, where ants such as *Technomyrmex jocosus* Forel, 1910 and *Messor barbarus* (Linnaeus, 1767) are parasitized by *Epacteon latifrons* Brown & Oliver, 2014 and *Razorfemora zaragozae* Disney, 2006, respectively; predation, where the phorid *Aphiura breviceps* Schmitz, 1939 preys on the eggs of the frog *Heleioporus albopunctatus* Gray, 1841 (Davis et al. 2003); and kleptoparasitism, as seen with *P. kerteszi*, which invades the nests of stingless bees like *Melipona fasciculata* Smith, 1854 to lay eggs in their pollen pots (Oliveira et al. 2013). In contrast to these specialists, genera like *Megaselia* showed lower *d*′ values, reflecting a broader host range.

The generalist nature of *Megaselia* is well established far beyond the scope of this review; its species are common subjects of study in fields as diverse as mushroom cultivation, where they are known pests, and forensic entomology, where they are important in decomposition timelines (White 1987; Manlove and Disney 2008; Brown et al. 2017). This ubiquity supports its characterization as a highly successful generalist. Finally, the results for some genera must be interpreted with caution, as the literature search may not have captured their complete host range. *P. kerteszi* serves as a prime example. While our network analysis linked it to only a few hosts, it is widely known to be a generalist kleptoparasite of many stingless bee species. Its documented hosts also include *Melipona beecheii* Bennett, 1831, *Melipona scutellaris* Latreille, 1811, *Melipona mandacaia* Smith, 1863, *Scaptotrigona postica* (Latreille, 1807), and even the honey bee *A. mellifera*, among others (Robinson 1981; Nogueira-Neto 1997; Robroek et al. 2003; Correia et al. 2024). This discrepancy highlights how network metrics are sensitive to the breadth of the underlying data and underscores the importance of integrating broader ecological knowledge when interpreting such results.

Importantly, because this network is derived from literature records, interaction frequencies likely reflect research effort rather than ecological abundance, and should therefore be interpreted with caution.

## Chemical Ecology Involved in the Interactions

Studying chemical ecology in host–parasite interactions is an important approach for comprehending how phorids locate their hosts. These mechanisms may influence patterns of host specialization observed in ecological networks. Among the accessed studies, only 14 articles refer to this theme, corresponding to 9.27% of the dataset. Therefore, conclusions regarding chemical mediation in phorid–host interactions should be interpreted with caution. The available information referring to chemical ecology is summarized in Table 1.

**Table 1:** List of Phoridae species, hosts, and synthesis of information about chemical ecology in each article

Phoridae sp.	Host sp.	Chemical ecology	Attractants compounds	References
*Ap. paraponerae* Borgmeier, 1958	*P. clavata* (Fabricius, 1775)	Attraction to the host body extract	Not specified	Brown and Feener Jr (1991)
*Pseudacteon tricuspis* Borgmeier, 1925	*S. invicta*	Attraction to live hosts and host body extract	Not specified	Chen and Fadamiro (2007)
*Pseudacteon cultellatus* Borgmeier, 1925; *Pseudacteon curvatus* Borgmeier, 1925; *Pseudacteon obtusus* Borgmeier, 1925; and *P. tricuspis*	*Solenopsis* spp.	Attraction to the alarm pheromone isomer and two analogs	2-ethyl-3,6(or 5)-dimethyl pyrazines; 2,3-diethyl-5-methyl pyrazine; 2-ethyl-3,5 + 6 methyl pyrazine	Ngumbi and Fadamiro (2015)
*P. tricuspis*, *P. curvatus*	*Solenopsis richteri* Forel, 1909, *S. invicta*, and *S. richteri* × *S. invicta* (hybrid)	Attraction to the host body extract	Not specified	Chen et al. (2012)
*P. lasciniosus*	*Az.sericeasur*	Cuticular hydrocarbons as host recognition cues	Straight chain alkanes, monomethyl alkanes, and dimethyl alkanes	Mathis and Tsutsui (2016)
*P. tricuspis*, *P. obtusus*, and *P. curvatus*	*S. invicta*	Attraction to a mixture of alarm pheromone and venom alkaloids	2-ethyl-3,6(or 5)-dimethyl pyrazines	Sharma and Fadamiro (2013)
*P. tricuspis*	*S. invicta*	Physiological response to venom alkaloid fractions	2,6-dialkylpiperidines	Chen et al. (2009)
*P. tricuspis*	*S. invicta*	Attraction to host worker alarm pheromones and other defensive compounds	Not specified	Morrison and King (2004)
*P. curvatus* and *P. obtusus*	*S. invicta*, *S. richteri*, and *Solenopsis geminata* (Fabricius, 1804)	Attraction to host venom alkaloid mixture	2-methyl-6-alkylpiperidines	Ajayi et al. (2020)
*Neodohrniphora elongata* Brown, 2001	*Atta sexdens* (Linnaeus, 1758)	Trail pheromone associated with visualization as host recognition cues	Not specified	Gazal et al. (2009)
*Myriophora communis* Hash & Brown, 2015; *Myriophora* sp. Brown, 1992	*Narceus americanus* (Palisot de Beauvouis, 1817); *Leptogoniulus sorornus* (Butler, 1876)	Attraction to defensive secretion	2-methoxy-3-methyl-1,4-benzoquinone	Hash et al. (2017)
*Pseudacteon* sp. Coquillett, 1907	*Azteca instabilis* (Smith, 1862)	Attraction to the pygidial gland	1-acetyl-2-methylcyclopentane	Mathis et al. (2011)
*P. tricuspis*	*S. invicta*	Physiological and behavioral response to alarm pheromone	2-ethyl-3,6(or 5)-dimethyl pyrazines	Sharma et al. (2011)
*Ap. paraponerae*	*P. clavata*	Attraction to the products of the mandibular glands that serve as alarm pheromones	4-methyl-3-heptanone; 4-methyl-3-heptanol	Feener et al. (1996)

Semiochemicals are chemical signals that mediate interactions between organisms, serving functions like species recognition, alarm signaling, and trail marking (Howard and Blomquist 1982). Phorid flies exploit this chemical landscape by responding to host-derived compounds during host location and recognition (Morehead and Feener 2000). Research has shown that these flies can exploit a wide variety of chemical cues, grouped into (i) pheromones used in host communication (e.g., trail and alarm pheromones), (ii) glandular secretions (e.g., venom alkaloids and mandibular or pygidial gland compounds), and (iii) general body-derived cues, including cuticular hydrocarbons and whole-body extracts (Feener et al. 1996; Morrison and King 2004; Sharma et al. 2011; Mathis and Tsutsui 2016).

Whole-body extracts are frequently reported as attractive cues for phorid flies; however, their chemical composition is often not fully characterized. Therefore, although cuticular hydrocarbons may be present in these extracts, their specific role cannot be assumed without direct experimental evidence. For example, *Ap. paraponerae* is more strongly attracted to extracts of its host *P. clavata* than to extracts from other ant species, suggesting a degree of host-associated chemical specificity, although the active compounds remain unidentified (Brown and Feener Jr 1991).

Experimental evidence suggests that different types of chemical cues may operate at different spatial scales. In the interaction between *Pseudacteon tricuspis* and *Solenopsis invicta*, flies respond strongly to crude body extracts, while isolated components such as (E,E)-α-farnesene do not elicit significant antennal responses (Chen and Fadamiro 2007). This indicates that complex odor blends, rather than single compounds, may be important for host location in some systems.

Phorid attraction is not restricted to disturbance-related cues. Although several studies report strong responses to alarm pheromones and defensive compounds released during colony disturbance (Morrison and King 2004), other systems demonstrate attraction to hosts during routine activities, such as foraging or recruitment (Almeida et al. 2021). This variation suggests that host-location mechanisms differ across taxa and ecological contexts.

The process of host location often involves multiple sensory modalities. In the interaction between *Pseudacteon lasciniosus* and *Azteca sericeasur*, flies are initially attracted by volatile cues and subsequently rely on visual and contact-based signals, such as cuticular hydrocarbons, to confirm host identity (Mathis and Tsutsui 2016). This sequential use of cues highlights the complexity of host recognition mechanisms.

The use of host alarm pheromones as cues for host location has been well documented in several phorid systems, particularly in interactions involving fire ants and *Pseudacteon* species. For example, both male and female *Apocephalus paraponerae* are attracted to specific compounds released from the mandibular glands of their giant ant host, *Paraponera clavata*. These compounds, including 4-methyl-3-heptanone and 4-methyl-3-heptanol, function as alarm pheromones for the ants during colony disturbances and are exploited by the flies as host-location cues (Feener et al. 1996). Similarly, research has identified the chemical signals guiding *Pseudacteon* flies, with electroantennogram and olfactometer experiments confirming that females of *P. tricuspis* respond to the fire ant alarm pheromone isomer 2-ethyl-3,6(or 5)-dimethyl pyrazine (Sharma et al. 2011). This response is not restricted to a single species; *P. cultellatus*, *P. obtusus*, and *P. curvatus* also respond to this compound. Furthermore, these flies can detect structurally similar alkylpyrazine analogs, although these are generally less attractive than the natural pheromone. These findings indicate that, while phorid flies can exhibit highly specific responses to host-derived chemical signals, the use of alarm pheromones as host-location cues appears to be taxon-specific rather than universal across Phoridae. This pattern also has implications for the development of biological control strategies targeting fire ants (Ngumbi and Fadamiro 2015).

The chemical cues involved in host location are diverse and may vary in their relative importance across systems. While cuticular hydrocarbons are often considered potential candidates for host recognition due to their role in insect communication, experimental evidence indicates that other compounds can play a more prominent role. For example, electroantennogram assays with *Pseudacteon tricuspis* demonstrated that responses to abdominal extracts, particularly those containing venom alkaloids, were significantly stronger than responses to head extracts associated with alarm pheromones (Chen et al. 2009). These findings suggest that venom-derived compounds may represent key cues for host detection in this system, potentially overriding other chemical signals such as cuticular hydrocarbons. More broadly, this highlights that phorid flies may rely on different components of the host chemical profile depending on the species and ecological context, reinforcing the need to avoid generalizations about the primary cues involved in host location. Furthermore, mixtures of alarm pheromones and venom alkaloids have been shown to produce stronger attraction than isolated compounds, indicating synergistic effects (Sharma and Fadamiro 2013).

Chemical cues may also mediate host specificity. For example, *P. curvatus* and *P. obtusus* show stronger attraction to venom alkaloid profiles of invasive fire ants than to those of native species, which may contribute to host preference patterns (Ajayi et al. 2020). Similarly, attraction to pygidial gland compounds has been demonstrated in other ant–phorid systems (Mathis et al. 2011). Also, in some interactions, chemical cues act in combination with visual stimuli. For example, in the association between *Neodohrniphora elongata* and *Atta sexdens*, visual cues such as host movement play a primary role, while chemical cues enhance host recognition at short range (Gazal et al. 2009). This reinforces the importance of multimodal integration in host location.

Finally, some phorid flies exploit defensive compounds produced by their hosts as kairomones. *Hiltonius millipedes*, for example, produce defensive secretions containing volatile quinones to repel predators. For *Myriophora* parasitoid flies, however, these same defensive compounds function as a reliable, long-range beacon. The presence of just one compound, 2-methoxy-3-methyl-1,4-benzoquinone, is enough to attract the flies, but a mixture of the two primary quinones is synergistically more attractive, turning the millipede’s primary defense into a potent and specific attractant for its enemy (Hash et al. 2017).

Overall, available studies indicate that chemical cues play an important role in mediating phorid–host interactions. However, the limited number of studies and the strong bias toward particular host systems restrict broader generalizations. Further research across a wider range of taxa is necessary to determine how widespread these mechanisms are within Phoridae.

## Behavioral Biology Among Phorid–Host Interactions

Behavioral strategies are often closely associated with the chemical cues involved in host detection. Approximately 30% of the reviewed articles described behavioral aspects of phorid–host interactions, with parasitoidism representing the dominant strategy (~92% of these studies). The genus *Pseudacteon* was the most frequently studied (31%), followed by *Apocephalus* (9%), and ants were the primary hosts (76%), reflecting the overall bias observed in the literature.

Based on the concepts of behavioral analysis (Del Claro 2004), phorid–host interactions involve a sequence of behavioral stages that include host location, inspection (e.g., hovering or short flights), selective oviposition, resource exploitation, and host defensive responses. Although these stages are often described separately, they are functionally integrated and may vary across taxa and ecological contexts.

### Host Location and Inspection Behavior

Host location typically begins with inspection flights, during which phorid flies hover around potential hosts. This behavior is likely mediated by a combination of visual and chemical cues, although the relative importance of these signals may vary across systems. For example, *Pseudacteon solenopsidis* exhibits characteristic hovering and pursuit behavior around fire ants, including backward flight and extended patrolling of foraging trails (Orr et al. 1997).

Inspection behavior generally precedes oviposition and allows flies to assess host suitability based on characteristics such as size, caste, sex, and location. This selectivity is evident in parasitoids such as *Apocephalus borealis* Brues, 1924, which affects more males than females of *Bombus* spp. (Otterstatter et al. 2002). Attacks are typically rapid; for instance, *A. borealis* females pursue honey bee workers, land on their abdomens, and insert their ovipositors within seconds (Core et al. 2012). Attack rates may also vary with host activity, as *Pseudacteon* species preferentially target *Solenopsis* ants at trails, feeding sites, and mound entrances, with higher attack frequencies associated with increased host movement (Folgarait and Gilbert 1999).

### Host Selection and Oviposition

Host selection in phorid parasitoids involves multiple factors, including host size, caste, sex, and behavioral context. Rather than reflecting active “choice” in a strict sense, these patterns likely emerge from constraints related to successful larval development and oviposition success. For instance, *Apocephalus borealis* has been reported more frequently in male *Bombus* hosts than in females (Otterstatter et al. 2002), although the underlying mechanisms remain unclear.

Host size is one of the most commonly reported factors influencing oviposition. Larger workers are often targeted, as observed in *Apocephalus* spp. and *Eibesfeldtphora* spp., likely because they provide sufficient resources for larval development (Feener Jr 1981; Guillade and Folgarait 2011). However, other species, such as *Myrmosicarius grandicornis*, preferentially attack smaller or foraging workers, indicating that host size preference varies among taxa and ecological strategies (Tonhasca et al. 2001).

Host selection may also occur at a finer scale, including specific body regions used for oviposition. In several systems, oviposition occurs in structurally vulnerable regions such as sutures, intersegmental membranes, or specific anatomical openings (Feener Jr and Brown 1993; Pereira et al. 2022). In some cases, females perform detailed inspection behaviors prior to oviposition, including walking on the host body and contacting specific regions before egg deposition.

In systems where multiple parasitoid species exploit the same host, resource partitioning can occur. Different phorid species may specialize on distinct host sizes, tasks, or oviposition sites, reducing direct competition and allowing coexistence. For example, multiple phorid species attacking *Atta* ants exhibit differences in attack location and oviposition strategy (Bragança et al. 2003). In some cases, parasitoids preferentially target injured or compromised hosts, likely due to reduced defensive capacity or increased accessibility of internal tissues (Brown and Feener Jr 1991; El-Hawagry et al. 2021).

### Multimodal Cues and Resource Exploitation in Host Interactions

Behavioral responses during host location and oviposition are often mediated by the integration of multiple sensory cues. In several systems, chemical signals alone are insufficient to trigger oviposition, requiring the presence of visual or tactile stimuli. For example, in the interaction between *Neodohrniphora elongata* and *Atta sexdens*, visual cues such as host movement play a key role, while chemical signals enhance host recognition at short range (Gazal et al. 2009). Similarly, *Pseudacteon* flies attacking *Azteca instabilis* rely on a combination of pheromonal cues and host movement for successful oviposition (Mathis et al. 2011). These findings reinforce the importance of multimodal integration in phorid–host-location strategies.

Beyond host detection and oviposition, some phorid species exploit host resources in ways that extend beyond direct parasitism. These interactions include the use of brood, stored food, and nest structures. Kleptoparasitic behavior has been reported in genera such as *Megaselia*, where larvae consume host provisions and immature stages within nests of bees and wasps (Simões et al. 1996; Gonzalez et al. 2002). In some cases, infestations reach high larval densities and cause substantial damage to host colonies. In addition to kleptoparasitism, predatory behavior on host eggs has also been documented. Phorid larvae have been recorded developing within spider egg sacs and feeding on amphibian eggs (Guarisco 2001; Ament and dos Santos 2017). These patterns indicate that phorid flies can exploit a wide range of trophic resources.

Overall, the integration of multimodal cues during host location, combined with diverse strategies of resource exploitation, highlights the ecological versatility of Phoridae. These interactions extend beyond parasitoidism and encompass multiple ecological strategies, reinforcing the complexity of phorid–host relationships.

### Grooming and Chemical Camouflage

Some phorid species exhibit behaviors consistent with chemical camouflage. In the interaction between *Commoptera solenipsidis* and its ant host *Pheidole dentata*, flies perform a sequence of grooming behaviors that may facilitate the acquisition and redistribution of host chemical cues (Seid and Brown 2009). This process involves initial contact with the host, followed by allogrooming, during which the fly likely acquires colony-specific chemical compounds, and subsequent self-grooming that may redistribute these cues across its body.

This behavior is interpreted as a mechanism that may reduce detection by the host colony, allowing the parasitoid to move among workers with a lower risk of recognition. However, the effectiveness, specificity, and prevalence of this strategy across Phoridae remain poorly understood.

### Host Defensive Behavior and Ecological Consequences

Hosts exhibit a wide range of defensive responses to phorid attacks, including individual behaviors such as evasive movements, defensive postures, and direct aggression toward attacking flies. One of the most commonly reported responses is the “C” posture, observed in multiple ant species under phorid attack, including *Solenopsis invicta* and *Messor barbarus* (Wuellner et al. 2002; Delgado et al. 2020). In addition, behaviors such as accelerated walking, biting, and defensive aggregation have been documented, as well as more specialized strategies like hitchhiking, in which smaller workers ride on transported leaves to reduce exposure to aerial parasitoids (Vieira-Neto et al. 2006).

At the colony level, responses may include changes in foraging behavior, reduced recruitment, and, in extreme cases, nest abandonment (Simões et al. 1996; Core et al. 2012). These behavioral changes can have broader ecological consequences by altering competitive interactions among species. For example, reduced activity of dominant ants due to phorid pressure can facilitate resource access for subordinate species, thereby influencing community structure (Philpott 2005).

Phorid-induced behavioral changes may also affect colony efficiency. Reduced foraging activity, altered worker size distribution, and increased abandonment of food resources have been documented in several systems (Braganca et al. 1998; Guillade and Folgarait 2015). In some cases, colonies adjust their foraging strategies to minimize risk, such as by deploying smaller workers that fall below the size threshold for parasitoid oviposition (Orr 1992). Together, these findings indicate that phorid parasitoids can influence host behavior across multiple levels of biological organization, from individual defensive acts to colony dynamics and broader ecological interactions.

### Conceptual Framework of Phorid–Host Interactions

**Fig. 5:**
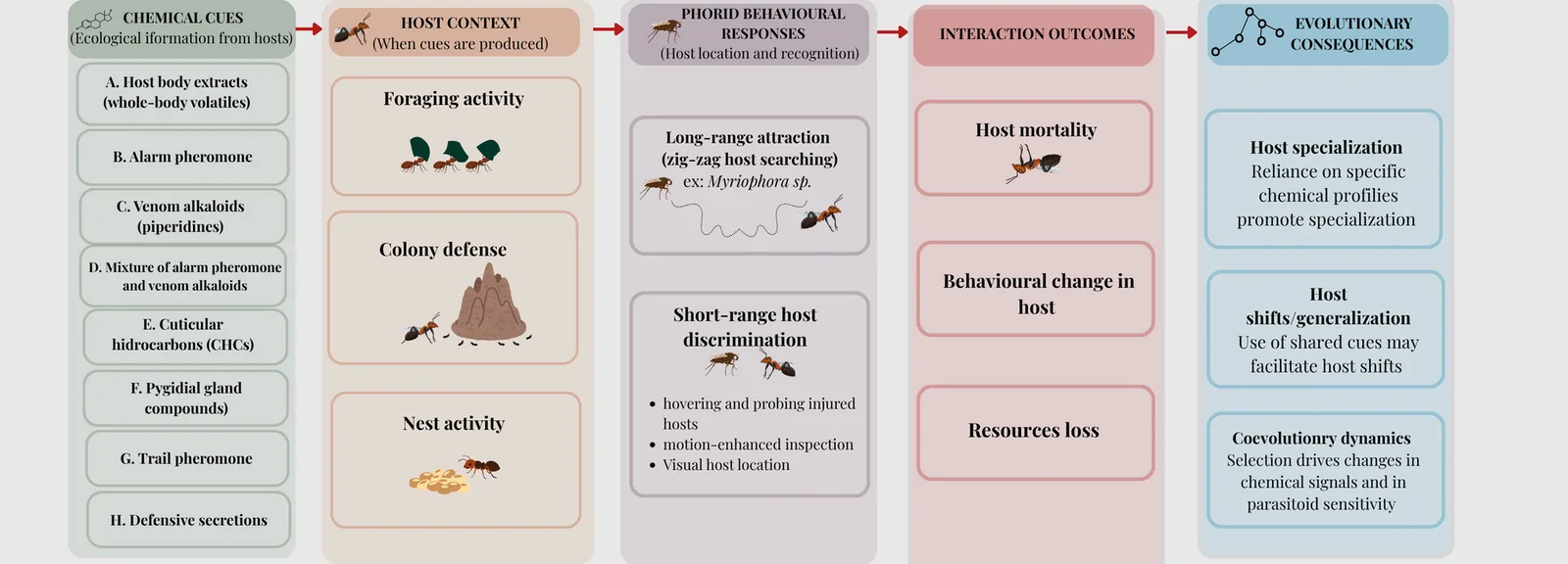
Conceptual model of chemical signal-mediated interactions between phorid flies and their hosts, connecting host-derived chemical cues, ecological context, parasitoid behavioral responses, parasitism outcomes, and their evolutionary consequences. Phorid fly vector used in the figure: photograph by Tom Murray, available under CC BY-ND-NC 1.0 license

To integrate the main patterns identified in the reviewed literature, we propose a conceptual framework linking chemical cues, host context, phorid behavioral responses, interaction outcomes, and their potential evolutionary consequences (Fig. 5). In this framework, host-derived chemical signals mediate parasitoid host location and recognition under different ecological contexts, shaping behavioral responses and influencing interaction outcomes such as host mortality, behavioral modification, and resource loss. Over evolutionary timescales, these interactions may promote host specialization, facilitate host shifts, and drive coevolutionary dynamics between phorids and their hosts. The framework also highlights current knowledge gaps, particularly the limited chemical characterization of many host–phorid systems compared with the more frequently documented behavioral interactions.

## Concluding Remarks

Parasitism is widespread in the Phoridae family and appears to have evolved multiple times, particularly within the Phorinae subfamily. Clarifying its evolutionary origins will require integrative approaches combining taxonomy, phylogeny, and behavioral data. Parasitoidism remains the most frequently reported interaction, whereas predation and kleptoparasitism are comparatively underrepresented, highlighting important gaps in our understanding of the ecological diversity of this group.

Ecological network analyses identified *Apocephalus* and *Megaselia* as central genera due to their associations with multiple hosts. However, these patterns are likely influenced by research bias, particularly the strong focus on ant-associated systems. Similarly, specialization patterns indicate that while some taxa, such as *Megaselia* and *Melaloncha*, exhibit generalist tendencies, others (e.g., the bee-associated kleptoparasite *Pseudohypocera*) show high levels of host specificity.

Chemical ecology has emerged as a key framework for understanding host location and selection, with semiochemicals, particularly cuticular hydrocarbons, alarm pheromones, and venom alkaloids, playing central roles. However, the limited number of studies and their concentration in a few model systems restrict broader generalizations. Importantly, evidence from both chemical and behavioral studies indicates that host location is often mediated by the integration of multiple sensory cues, rather than by single chemical compounds alone.

The behavioral sequence underlying phorid–host interactions typically involve host detection, inspection, and oviposition, but this process is highly variable across taxa and ecological contexts. In addition, interactions extend beyond parasitoidism to include kleptoparasitism, predation, and exploitation of host resources, reinforcing the ecological versatility of Phoridae.

Overall, the exceptional diversity of life histories within Phoridae makes this family a valuable model for investigating the evolution of parasitic and host-associated strategies. Future research integrating ecological networks, behavioral observations, and chemical analyses across a broader range of taxa will be essential to uncover the mechanisms driving host use and diversification in this group.

## Supplementary Information

Below is the link to the electronic supplementary material.ESM 1DOCX (982 KB)

## Data Availability

The authors declare that the data supporting the findings of this study are available within the paper and its Supplementary Information files. Should any raw data files be needed in another format, they are available from the corresponding author upon reasonable request.
