# Supplementary material for: Behavior and Chemical Ecology in Phoridae (Diptera): A Systematic Review of Parasitoid-Host Interactions
Source: Neotrop Entomol. 2026 Aug 31;55(1):80. doi: 10.1007/s13744-026-01431-5 (PMC13529873; doi:10.1007/s13744-026-01431-5)
Supplement: Supplementary file 1 [file 13744_2026_1431_MOESM1_ESM.docx]

**Table 1.** Phoridae species, associated host species, and interaction types reported in the systematic review of publications up to 2024.

| **Phoridae sp** | **Host sp** | **Interaction** | **Reference** |
| --- | --- | --- | --- |
| *Aphiura breviceps* Schmitz, 1939 | *Heleioporus albopunctatus* Gray, 1841 | Predation | Davis, Henry & Disney, 2003 |
| *Apocephalus (Mesophora) apivorus* Brown, 1996 | *Cephalotrigona capitata* (Smith, 1854) | Parasitoidism | Brown, 1997 |
| *Apocephalus antennatus* Malloch, 1913 | *Ellychnia corrusca* (Linnaeus, 1767) | Parasitoidism | Brown, 1994 |
| *Apocephalus antennatus* Malloch, 1913 | *Photinus marginellus* LeConte, 1852 | Parasitoidism | Brown, 1994 |
| *Apocephalus antennatus* Malloch, 1913 | *Lucidota atra* (Oliver, 1790) | Parasitoidism | Brown, 1994 |
| *Apocephalus antennatus* Malloch, 1913 | *Photinus marginellus* LeConte, 1852 | Parasitoidism | Brown, 1994 |
| *Apocephalus antennatus* Malloch, 1913 | *Photinus pyralis* (Linnaeus, 1767) | Parasitoidism | Brown, 1994 |
| *Apocephalus attophilus* Borgmeier, 1928 | *Atta bisphaerica* Forel, 1908 | Parasitoidism | Bragança, Della Lucia & Tonhasca Jr, 2003; Bragança et al., 2021; Farder-Gomes et al., 2018 |
| *Apocephalus attophilus* Borgmeier, 1928 | *Atta laevigata* (Smith, 1858) | Parasitoidism | Brown, 1994; Erthal & Tonhasca, 2000; Bragança, Zodja & Medeiros, 2006; Bragança et al., 2016; Arruda et al., 2019; Souza et al., 2021a; Pimentel et al., 2022; de Almeida et al., 2023 |
| *Apocephalus attophilus* Borgmeier, 1928 | *Atta sexdens* (Linnaeus, 1758) | Parasitoidism | Farder-Gomes et al., 2017, 2020; Arruda et al., 2019; Galvão et al., 2019; Pereira et al., 2022 |
| *Apocephalus borealis* Brues, 1924 | *Apis mellifera* Linnaeus, 1758 | Parasitoidism | Core et al., 2012a; Ravoet et al., 2013 |
| *Apocephalus borealis* Brues, 1924 | *Bombus bifarius* Cresson, 1879 | Parasitoidism | Otterstatter, Whidden & Owen, 2002 |
| *Apocephalus borealis* Brues, 1924 | *Bombus occidentalis* Greene, 1858 | Parasitoidism | Otterstatter, Whidden & Owen, 2002 |
| *Apocephalus borealis* Brues, 1924 | *Bombus vosnesenskii* Radoszkowski, 1862 | Parasitoidism | Core et al., 2012 |
| *Apocephalus borealis* Brues, 1924 | *Bombus flavifrons* Cresson, 1863 | Parasitoidism | Otterstatter, Whidden & Owen, 2002 |
| *Apocephalus borealis* Brues, 1924 | *Bombus melanopygus* Nylander, 1848 | Parasitoidism | Core et al., 2012 |
| *Apocephalus borealis* Brues, 1924 | *Bombus californicus* Smith, 1854 | Parasitoidism | Otterstatter, Whidden & Owen, 2002 |
| *Apocephalus exlobicornis* Brown & Disney, 2010 | *Acromyrmex crassispinus* (Forel, 1909) | Parasitoidism | Elizalde & Folgarait, 2011 |
| *Apocephalus exlobicornis* Brown & Disney, 2010 | *Acromyrmex fracticornis* (Forel, 1909) | Parasitoidism | Elizalde & Folgarait, 2011 |
| *Apocephalus exlobicornis* Brown & Disney, 2010 | *Acromyrmex heyeri* (Forel, 1899) | Parasitoidism | Elizalde & Folgarait, 2011 |
| *Apocephalus exlobicornis* Brown & Disney, 2010 | *Acromyrmex hispidus* Santschi, 1925 | Parasitoidism | Elizalde & Folgarait, 2011 |
| *Apocephalus exlobicornis* Brown & Disney, 2010 | *Acromyrmex lobicornis* (Emery, 1888) | Parasitoidism | Elizalde & Folgarait, 2011 |
| *Apocephalus exlobicornis* Brown & Disney, 2010 | *Acromyrmex lundii* (Guérin-Méneville, 1838) | Parasitoidism | Elizalde & Folgarait, 2011 |
| *Apocephalus exlobicornis* Brown & Disney, 2010 | *Acromyrmex striatus* (Roger, 1863) | Parasitoidism | Elizalde & Folgarait, 2011 |
| *Apocephalus exlobicornis* Brown & Disney, 2010 | *Atta vollenweideri* Forel, 1893 | Parasitoidism | Elizalde & Folgarait, 2011 |
| *Apocephalus exstriatus* Brown & Disney, 2010 | *Acromyrmex crassispinus* (Forel, 1909) | Parasitoidism | Elizalde & Folgarait, 2011 |
| *Apocephalus exstriatus* Brown & Disney, 2010 | *Acromyrmex fracticornis* (Forel, 1909) | Parasitoidism | Elizalde & Folgarait, 2011 |
| *Apocephalus exstriatus* Brown & Disney, 2010 | *Acromyrmex heyeri* (Forel, 1899) | Parasitoidism | Elizalde & Folgarait, 2011 |
| *Apocephalus exstriatus* Brown & Disney, 2010 | *Acromyrmex hispidus* Santschi, 1925 | Parasitoidism | Elizalde & Folgarait, 2011 |
| *Apocephalus exstriatus* Brown & Disney, 2010 | *Acromyrmex lobicornis* (Emery, 1888) | Parasitoidism | Elizalde & Folgarait, 2011 |
| *Apocephalus exstriatus* Brown & Disney, 2010 | *Acromyrmex lundii* (Guérin-Méneville, 1838) | Parasitoidism | Elizalde & Folgarait, 2011 |
| *Apocephalus exstriatus* Brown & Disney, 2010 | *Acromyrmex striatus* (Roger, 1863) | Parasitoidism | Elizalde & Folgarait, 2011 |
| *Apocephalus exstriatus* Brown & Disney, 2010 | *Atta vollenweideri* Forel, 1893 | Parasitoidism | Elizalde & Folgarait, 2011 |
| *Apocephalus intermedius* Brown & Disney, 2010 | *Acromyrmex crassispinus* (Forel, 1909) | Parasitoidism | Elizalde & Folgarait, 2011 |
| *Apocephalus intermedius* Brown & Disney, 2010 | *Acromyrmex fracticornis* (Forel, 1909) | Parasitoidism | Elizalde & Folgarait, 2011 |
| *Apocephalus intermedius* Brown & Disney, 2010 | *Acromyrmex heyeri* (Forel, 1899) | Parasitoidism | Elizalde & Folgarait, 2011 |
| *Apocephalus intermedius* Brown & Disney, 2010 | *Acromyrmex hispidus* Santschi, 1925 | Parasitoidism | Elizalde & Folgarait, 2011 |
| *Apocephalus intermedius* Brown & Disney, 2010 | *Acromyrmex lobicornis* (Emery, 1888) | Parasitoidism | Elizalde & Folgarait, 2011 |
| *Apocephalus intermedius* Brown & Disney, 2010 | *Acromyrmex lundii* (Guérin-Méneville, 1838) | Parasitoidism | Elizalde & Folgarait, 2011 |
| *Apocephalus intermedius* Brown & Disney, 2010 | *Acromyrmex striatus* (Roger, 1863) | Parasitoidism | Elizalde & Folgarait, 2011 |
| *Apocephalus intermedius* Brown & Disney, 2010 | *Atta vollenweideri* Forel, 1893 | Parasitoidism | Elizalde & Folgarait, 2011 |
| *Apocephalus longisetarum* Brown & Disney, 2010 | *Acromyrmex crassispinus* (Forel, 1909) | Parasitoidism | Elizalde & Folgarait, 2011 |
| *Apocephalus longisetarum* Brown & Disney, 2010 | *Acromyrmex fracticornis* (Forel, 1909) | Parasitoidism | Elizalde & Folgarait, 2011 |
| *Apocephalus longisetarum* Brown & Disney, 2010 | *Acromyrmex heyeri* (Forel, 1899) | Parasitoidism | Elizalde & Folgarait, 2011 |
| *Apocephalus longisetarum* Brown & Disney, 2010 | *Acromyrmex hispidus* Santschi, 1925 | Parasitoidism | Elizalde & Folgarait, 2011 |
| *Apocephalus longisetarum* Brown & Disney, 2010 | *Acromyrmex lobicornis* (Emery, 1888) | Parasitoidism | Elizalde & Folgarait, 2011 |
| *Apocephalus longisetarum* Brown & Disney, 2010 | *Acromyrmex lundii* (Guérin-Méneville, 1838) | Parasitoidism | Elizalde & Folgarait, 2011 |
| *Apocephalus longisetarum* Brown & Disney, 2010 | *Acromyrmex striatus* (Roger, 1863) | Parasitoidism | Elizalde & Folgarait, 2011 |
| *Apocephalus longisetarum* Brown & Disney, 2010 | *Atta vollenweideri* Forel, 1893 | Parasitoidism | Elizalde & Folgarait, 2011 |
| *Apocephalus lutheihalteratus* Borgmeier, 1923 | *Acromyrmex coronatus* (Fabricius, 1804) | Parasitoidism | Elizalde & Queiroz, 2013 |
| *Apocephalus neivai* Borgmeier, 1931 | *Acromyrmex ambiguus* (Emery, 1888) | Parasitoidism | Guillade, Goffré & Folgarait, 2017; Goffre & Folgarait, 2019 |
| *Apocephalus neivai* Borgmeier, 1931 | *Acromyrmex crassispinus* (Forel, 1909) | Parasitoidism | Elizalde & Folgarait, 2011; Guillade et al., 2017 |
| *Apocephalus neivai* Borgmeier, 1931 | *Acromyrmex fracticornis* (Forel, 1909) | Parasitoidism | Elizalde & Folgarait, 2011, 2012 |
| *Apocephalus neivai* Borgmeier, 1931 | *Acromyrmex heyeri* (Forel, 1899) | Parasitoidism | Elizalde & Folgarait, 2011, 2012; Guillade et al., 2017; Goffre & Folgarait, 2019 |
| *Apocephalus neivai* Borgmeier, 1931 | *Acromyrmex hispidus* Santschi, 1925 | Parasitoidism | Elizalde & Folgarait, 2011, 2012 |
| *Apocephalus neivai* Borgmeier, 1931 | *Acromyrmex lobicornis* (Emery, 1888) | Parasitoidism | Elizalde & Folgarait, 2011, 2012; Guillade et al., 2017 |
| *Apocephalus neivai* Borgmeier, 1931 | *Acromyrmex lundii* (Guérin-Méneville, 1838) | Parasitoidism | Elizalde & Folgarait, 2011, 2012; Guillade et al., 2017; Goffre & Folgarait, 2019 |
| *Apocephalus neivai* Borgmeier, 1931 | Acromyrmex sp. Mayr, 1865 | Parasitoidism | Elizalde et al., 2018 |
| *Apocephalus neivai* Borgmeier, 1931 | *Acromyrmex striatus* (Roger, 1863) | Parasitoidism | Elizalde & Folgarait, 2011, 2012 |
| *Apocephalus neivai* Borgmeier, 1931 | Atta sp. Fabricius, 1804 | Parasitoidism | Elizalde et al., 2018 |
| *Apocephalus neivai* Borgmeier, 1931 | *Atta vollenweideri* Forel, 1893 | Parasitoidism | Elizalde & Folgarait, 2011, 2012 |
| *Apocephalus noetingerorum* Brown & Disney, 2010 | *Acromyrmex ambiguus* (Emery, 1888) | Parasitoidism | Guillade et al., 2017; Goffre & Folgarait, 2019 |
| *Apocephalus noetingerorum* Brown & Disney, 2010 | *Acromyrmex fracticornis* (Forel, 1909) | Parasitoidism | Elizalde & Folgarait, 2011 |
| *Apocephalus noetingerorum* Brown & Disney, 2010 | *Acromyrmex heyeri* (Forel, 1899) | Parasitoidism | Elizalde & Folgarait, 2011; Goffre & Folgarait, 2019 |
| *Apocephalus noetingerorum* Brown & Disney, 2010 | *Acromyrmex hispidus* Santschi, 1925 | Parasitoidism | Elizalde & Folgarait, 2011 |
| *Apocephalus noetingerorum* Brown & Disney, 2010 | *Acromyrmex lobicornis* (Emery, 1888) | Parasitoidism | Elizalde & Folgarait, 2011 |
| *Apocephalus noetingerorum* Brown & Disney, 2010 | *Acromyrmex lundii* (Guérin-Méneville, 1838) | Parasitoidism | Elizalde & Folgarait, 2011; Guillade et al., 2017; Goffre & Folgarait, 2019 |
| *Apocephalus noetingerorum* Brown & Disney, 2010 | Acromyrmex sp. Mayr, 1865 | Parasitoidism | Elizalde et al., 2018 |
| *Apocephalus noetingerorum* Brown & Disney, 2010 | Atta sp. Fabricius, 1804 | Parasitoidism | Elizalde et al., 2018 |
| *Apocephalus noetingerorum* Brown & Disney, 2010 | *Atta vollenweideri* Forel, 1893 | Parasitoidism | Elizalde & Folgarait, 2011 |
| *Apocephalus noetingerorum* Brown & Disney, 2010 | *Acromyrmex crassispinus* (Forel, 1909) | Parasitoidism | Elizalde & Folgarait, 2011; Guillade et al., 2017 |
| *Apocephalus noetingerorum* Brown & Disney, 2010 | *Acromyrmex striatus* (Roger, 1863) | Parasitoidism | Elizalde & Folgarait, 2011 |
| *Apocephalus paraponerae* Borgmeier, 1958 | *Paraponera clavata* (Fabricius, 1775) | Parasitoidism | Brown & Feener, 1991a, 1991b; Feener, Jacobs & Schmidt, 1996 |
| *Apocephalus penicillatus* Brown & Disney, 2010 | *Acromyrmex crassispinus* (Forel, 1909) | Parasitoidism | Elizalde & Folgarait, 2011 |
| *Apocephalus penicillatus* Brown & Disney, 2010 | *Acromyrmex fracticornis* (Forel, 1909) | Parasitoidism | Elizalde & Folgarait, 2011 |
| *Apocephalus penicillatus* Brown & Disney, 2010 | *Acromyrmex heyeri* (Forel, 1899) | Parasitoidism | Elizalde & Folgarait, 2011 |
| *Apocephalus penicillatus* Brown & Disney, 2010 | *Acromyrmex hispidus* Santschi, 1925 | Parasitoidism | Elizalde & Folgarait, 2011 |
| *Apocephalus penicillatus* Brown & Disney, 2010 | *Acromyrmex lobicornis* (Emery, 1888) | Parasitoidism | Elizalde & Folgarait, 2011 |
| *Apocephalus penicillatus* Brown & Disney, 2010 | *Acromyrmex lundii* (Guérin-Méneville, 1838) | Parasitoidism | Elizalde & Folgarait, 2011 |
| *Apocephalus penicillatus* Brown & Disney, 2010 | Acromyrmex sp. Mayr, 1865 | Parasitoidism | Elizalde et al., 2018 |
| *Apocephalus penicillatus* Brown & Disney, 2010 | *Acromyrmex striatus* (Roger, 1863) | Parasitoidism | Elizalde & Folgarait, 2011 |
| *Apocephalus penicillatus* Brown & Disney, 2010 | Atta sp. Fabricius, 1804 | Parasitoidism | Elizalde et al., 2018 |
| *Apocephalus penicillatus* Brown & Disney, 2010 | *Atta vollenweideri* Forel, 1893 | Parasitoidism | Elizalde & Folgarait, 2011 |
| *Apocephalus philhispidus* Brown & Disney, 2010 | *Acromyrmex crassispinus* (Forel, 1909) | Parasitoidism | Elizalde & Folgarait, 2011 |
| *Apocephalus philhispidus* Brown & Disney, 2010 | *Acromyrmex fracticornis* (Forel, 1909) | Parasitoidism | Elizalde & Folgarait, 2011 |
| *Apocephalus philhispidus* Brown & Disney, 2010 | *Acromyrmex heyeri* (Forel, 1899) | Parasitoidism | Elizalde & Folgarait, 2011 |
| *Apocephalus philhispidus* Brown & Disney, 2010 | *Acromyrmex hispidus* Santschi, 1925 | Parasitoidism | Elizalde & Folgarait, 2011 |
| *Apocephalus philhispidus* Brown & Disney, 2010 | *Acromyrmex lobicornis* (Emery, 1888) | Parasitoidism | Elizalde & Folgarait, 2011 |
| *Apocephalus philhispidus* Brown & Disney, 2010 | *Acromyrmex lundii* (Guérin-Méneville, 1838) | Parasitoidism | Elizalde & Folgarait, 2011 |
| *Apocephalus philhispidus* Brown & Disney, 2010 | *Acromyrmex striatus* (Roger, 1863) | Parasitoidism | Elizalde & Folgarait, 2011 |
| *Apocephalus philhispidus* Brown & Disney, 2010 | *Atta vollenweideri* Forel, 1893 | Parasitoidism | Elizalde & Folgarait, 2011 |
| *Apocephalus setitarsus* Brown, 1997 | *Acromyrmex crassispinus* (Forel, 1909) | Parasitoidism | Elizalde & Folgarait, 2011 |
| *Apocephalus setitarsus* Brown, 1997 | *Acromyrmex fracticornis* (Forel, 1909) | Parasitoidism | Elizalde & Folgarait, 2011 |
| *Apocephalus setitarsus* Brown, 1997 | *Acromyrmex heyeri* (Forel, 1899) | Parasitoidism | Elizalde & Folgarait, 2011 |
| *Apocephalus setitarsus* Brown, 1997 | *Acromyrmex hispidus* Santschi, 1925 | Parasitoidism | Elizalde & Folgarait, 2011 |
| *Apocephalus setitarsus* Brown, 1997 | *Acromyrmex lobicornis* (Emery, 1888) | Parasitoidism | Elizalde & Folgarait, 2011 |
| *Apocephalus setitarsus* Brown, 1997 | *Acromyrmex lundii* (Guérin-Méneville, 1838) | Parasitoidism | Elizalde & Folgarait, 2011 |
| *Apocephalus setitarsus* Brown, 1997 | *Acromyrmex striatus* (Roger, 1863) | Parasitoidism | Elizalde & Folgarait, 2011 |
| *Apocephalus setitarsus* Brown, 1997 | *Atta vollenweideri* Forel, 1893 | Parasitoidism | Elizalde & Folgarait, 2011; Guillade & Folgarait, 2011 |
| *Apocephalus* sp. | *Atta cephalotes* (Linnaeus, 1758) | Parasitoidism | Uribe et al., 2016 |
| *Apocephalus* sp. | *Pheidole dentata* Mayr, 1886 | Parasitoidism | Feener Jr, 1981 |
| *Apocephalus* sp. | *Solenopsis texana* Emery, 1895 | Parasitoidism | Feener Jr, 1981 |
| *Apocephalus* spp. | *Acromyrmex niger* (Smith, 1858) | Parasitoidism | Barrera et al., 2017 |
| *Apocephalus* spp. | *Pheidole diversipilosa* Wheeler, 1908 | Parasitoidism | LeBrun & Feener, 2002, 2007; LeBrun, 2005 |
| *Apocephalus* spp. | *Pheidole bicarinata* Mayr, 1871 | Parasitoidism | LeBrun, 2005; LeBrun & Feener, 2007 |
| *Apocephalus tritarsus* Brown, 1993 | *Atalantycha neglecta* (Fall, 1919) | Parasitoidism | Brown, Faust & Cicero, 2009 |
| *Apocephalus vicosae* Disney, 2000 | *Acromyrmex crassispinus* (Forel, 1909) | Parasitoidism | Elizalde & Folgarait, 2011 |
| *Apocephalus vicosae* Disney, 2000 | *Acromyrmex fracticornis* (Forel, 1909) | Parasitoidism | Elizalde & Folgarait, 2011 |
| *Apocephalus vicosae* Disney, 2000 | *Acromyrmex heyeri* (Forel, 1899) | Parasitoidism | Elizalde & Folgarait, 2011 |
| *Apocephalus vicosae* Disney, 2000 | *Acromyrmex hispidus* Santschi, 1925 | Parasitoidism | Elizalde & Folgarait, 2011 |
| *Apocephalus vicosae* Disney, 2000 | *Acromyrmex lobicornis* (Emery, 1888) | Parasitoidism | Elizalde & Folgarait, 2011 |
| *Apocephalus vicosae* Disney, 2000 | *Acromyrmex lundii* (Guérin-Méneville, 1838) | Parasitoidism | Elizalde & Folgarait, 2011 |
| *Apocephalus vicosae* Disney, 2000 | *Acromyrmex striatus* (Roger, 1863) | Parasitoidism | Elizalde & Folgarait, 2011 |
| *Apocephalus vicosae* Disney, 2000 | *Atta laevigata* (Smith, 1858) | Parasitoidism | Bragança et al., 2006, 2016; Arruda et al., 2019; Souza et al., 2021b; Pimentel et al., 2022 |
| *Apocephalus vicosae* Disney, 2000 | *Atta sexdens* (Linnaeus, 1758) | Parasitoidism | Bragança et al., 2016; Arruda et al., 2019; Galvão et al., 2019 |
| *Apocephalus vicosae* Disney, 2000 | *Atta vollenweideri* Forel, 1893 | Parasitoidism | Elizalde & Folgarait, 2011 |
| *Apterophora* sp. | *Atta cephalotes* (Linnaeus, 1758) | Parasitoidism | Uribe et al., 2016 |
| *Auxanommatidia* sp. | *Camponotus morosus* (Smith, 1858) | Parasitoidism | Segura & Brown, 2014 |
| *Chonocephalus* sp. | *Atta cephalotes* (Linnaeus, 1758) | Parasitoidism | Uribe et al., 2016 |
| *Commoptera solenopsidis* Brues, 1901 | *Pheidole dentata* Mayr, 1886 | Parasitoidism | Seid & Brown, 2009 |
| *Coniceromyia* spp. | *Atta cephalotes* (Linnaeus, 1758) | Parasitoidism | Uribe et al., 2016 |
| *Cremersia adunca* Borgmeier, 1961 | *Neivamyrmex texanus* Watkins, 1972 | Parasitoidism | Jenkins, 2013 |
| *Cremersia* sp. | *Labidus praedator* (Smith, 1858) | Parasitoidism | Brown & Feener, 1998 |
| *Cremersia* sp. | *Neivamyrmex nigrescens* (Cresson, 1872) | Parasitoidism | Brown & Feener, 1998 |
| *Cremersia* sp. | *Nomamyrmex hartigii* (Westwood, 1842) | Parasitoidism | Brown & Feener, 1998 |
| *Dacnophora pectinatus* Brown, 1988 | *Labidus praedator* (Smith, 1858) | Parasitoidism | Brown & Feener, 1998 |
| *Dacnophora pectinatus* Brown, 1988 | *Neivamyrmex nigrescens* (Cresson, 1872) | Parasitoidism | Brown & Feener, 1998 |
| *Dacnophora pectinatus* Brown, 1988 | *Nomamyrmex hartigii* (Westwood, 1842) | Parasitoidism | Brown & Feener, 1998 |
| *Dacnophora* sp. | *Labidus praedator* (Smith, 1858) | Parasitoidism | Brown & Feener, 1998 |
| *Dacnophora* sp. | *Neivamyrmex nigrescens* (Cresson, 1872) | Parasitoidism | Brown & Feener, 1998 |
| *Dacnophora* sp. | *Nomamyrmex hartigii* (Westwood, 1842) | Parasitoidism | Brown & Feener, 1998 |
| *Dohrniphora* spp. | *Atta cephalotes* (Linnaeus, 1758) | Parasitoidism | Uribe et al., 2016 |
| *Eibesfeldtphora attae* (Disney, 1996) | *Atta cephalotes* (Linnaeus, 1758) | Parasitoidism | Uribe et al., 2016 |
| *Eibesfeldtphora* *bragancai* (Brown, 2001) | *Atta bisphaerica* Forel, 1908 | Parasitoidism | Farder-Gomes et al., 2018; Bragança et al., 2021 |
| *Eibesfeldtphora bragancai* (Brown, 2001) | *Atta bisphaerica* Forel, 1908 | Parasitoidism | Farder-Gomes et al., 2018 |
| *Eibesfeldtphora bragancai* (Brown, 2001) | *Atta laevigata* (Smith, 1858) | Parasitoidism | Pimentel et al., 2022 |
| *Eibesfeldtphora bragancai* (Brown, 2001) | *Atta sexdens* (Linnaeus, 1758) | Parasitoidism | Galvão et al., 2019 |
| *Eibesfeldtphora breviloba* Brown, 2013 | *Atta robusta* Borgmeier, 1939 | Parasitoidism | Brown et al., 2012 |
| *Eibesfeldtphora curvinervis* Borgmeier, 1962 | *Atta cephalotes* (Linnaeus, 1758) | Parasitoidism | Alma, Farji-Brener & Elizalde, 2019 |
| *Eibesfeldtphora declinata* (Bogmeier, 1925) | *Atta laevigata* (Smith, 1858) | Parasitoidism | Pimentel et al., 2022; de Almeida et al., 2023 |
| *Eibesfeldtphora digitata* Brown & Kung, 2007 | *Atta robusta* Borgmeier, 1939 | Parasitoidism | Brown et al., 2012 |
| *Eibesfeldtphora elongata* (Brown, 2001) | *Atta laevigata* (Smith, 1858) | Parasitoidism | de Almeida et al., 2023 |
| *Eibesfeldtphora erthali* (Brown, 2001) | *Atta laevigata* (Smith, 1858) | Parasitoidism | Arruda et al., 2019; Souza et al., 2021b; Pimentel et al., 2022 |
| *Eibesfeldtphora* sp. | *Atta vollenweideri* Forel, 1893 | Parasitoidism | Guillade & Folgarait, 2015 |
| *Eibesfeldtphora* spp. | *Atta laevigata* (Smith, 1858) | Parasitoidism | Bragança et al., 2016; Souza & Pereira, 2020 |
| *Eibesfeldtphora* spp. | *Atta sexdens* (Linnaeus, 1758) | Parasitoidism | Bragança et al., 2016; Souza & Pereira, 2020; Pereira et al., 2022 |
| *Eibesfeldtphora tonhascai* (Brown, 2001) | *Atta laevigata* (Smith, 1858) | Parasitoidism | Pereira et al., 2022; de Almeida et al., 2023 |
| *Eibesfeldtphora* *tonhascai* (Brown, 2001) | *Atta sexdens* (Linnaeus, 1758) | Parasitoidism | Farder-Gomes et al., 2017, 2020a; Galvão et al., 2019; Pereira et al., 2022 |
| *Eibesfeldtphora trilobata* Disney, 2009 | *Acromyrmex crassispinus* (Forel, 1909) | Parasitoidism | Elizalde & Folgarait, 2011, 2012 |
| *Eibesfeldtphora trilobata* Disney, 2009 | *Acromyrmex fracticornis* (Forel, 1909) | Parasitoidism | Elizalde & Folgarait, 2011, 2012 |
| *Eibesfeldtphora trilobata* Disney, 2009 | *Acromyrmex heyeri* (Forel, 1899) | Parasitoidism | Elizalde & Folgarait, 2011, 2012 |
| *Eibesfeldtphora trilobata* Disney, 2009 | *Acromyrmex hispidus* Santschi, 1925 | Parasitoidism | Elizalde & Folgarait, 2011, 2012 |
| *Eibesfeldtphora trilobata* Disney, 2009 | *Acromyrmex lobicornis* (Emery, 1888) | Parasitoidism | Elizalde & Folgarait, 2011, 2012 |
| *Eibesfeldtphora trilobata* Disney, 2009 | *Acromyrmex lundii* (Guérin-Méneville, 1838) | Parasitoidism | Elizalde & Folgarait, 2011, 2012 |
| *Eibesfeldtphora trilobata* Disney, 2009 | *Acromyrmex striatus* (Roger, 1863) | Parasitoidism | Elizalde & Folgarait, 2011, 2012 |
| *Eibesfeldtphora trilobata* Disney, 2009 | *Atta vollenweideri* Forel, 1893 | Parasitoidism | Elizalde & Folgarait, 2011, 2012; Guillade & Folgarait, 2011 |
| *Epacteon latifrons* Brown & Oliver, 2014 | *Technomyrmex jocosus* Forel, 1910 | Parasitoidism | Brown & Oliver, 2014 |
| Eurycnemis sp. Borgmeier, 1971 | *Atta cephalotes* (Linnaeus, 1758) | Parasitoidism | Uribe et al., 2016 |
| *Lucianaphora folgaraitae* Disney, 2008 | Acromyrmex sp. Mayr, 1865 | Parasitoidism | Elizalde et al., 2018 |
| *Lucianaphora folgaraitae* Disney, 2008 | Atta sp. Fabricius, 1804 | Parasitoidism | Elizalde et al., 2018 |
| *Megaselia bruchiana* Borgmeier & Schmitz, 1923 | *Phyllomedusa iheringii* Boulenger, 1885 | Predation | Ament & dos Santos, 2017 |
| *Megaselia necrophaga* Enderlein, 1912 | *Phyllomedusa iheringii* Boulenger, 1885 | Predation | Ament & dos Santos, 2017 |
| *Megaselia neocorynurae* Gonzalez, Brown & Ospina, 2002 | Neocorynura spp. Schrottky, 1879 | Kleptoparasitism | Gonzalez, Brown & Ospina, 2002 |
| *Megaselia picta* (Lehmann, 1822) | *Protopolybia exigua* (Saussure, 1854) | Parasitism | Simões, Noll & Zucchi, 1996 |
| *Megaselia rufipes* (Meigen, 1804) | *Apis mellifera* Linnaeus, 1758 | Parasitoidism | Dutto & Ferrazzi, 2014 |
| *Megaselia rufipes* (Meigen, 1804) | *Amphipyra glabella* Morrison, 1874 | Parasitoidism | Mccabe, 1998 |
| *Megaselia scalaris* (Loew, 1866) | *Agalychnis annae* (Duellman, 1963) | Predation | Villa & Townsend, 1983 |
| *Megaselia scalaris* (Loew, 1866) | *Apis mellifera* Linnaeus, 1758 | Parasitoidism | Menail et al., 2016; Ricchiuti et al., 2016 |
| *Megaselia scalaris* (Loew, 1866) | *Brachypelma vagans* (Ausserer, 1875) | Parasitoidism | MacHkour-M’Rabet, Dor & Hénaut, 2015 |
| *Megaselia scalaris* (Loew, 1866) | *Eleutherodactylus coqui* Thomas, 1967 | Predation | Villa & Townsend, 1983 |
| *Megaselia scalaris* (Loew, 1866) | *Hypsiboas caingua* Carrizo, 1991 | Parasitoidism | López et al., 2016 |
| *Megaselia scalaris* (Loew, 1866) | *Macrochelys temminckii* Troost, 1835 | Parasitoidism | Holcomb & Carr, 2011 |
| *Megaselia scalaris* (Loew, 1866) | *Nezara viridula* (Linnaeus, 1758) | Parasitoidism | El-Hawagry, Ebrahim & Nada, 2021 |
| *Megaselia scalaris* (Loew, 1866) | *Polybia occidentalis* (Olivier, 1791) | Parasitoidism | London & Jeanne, 1998 |
| *Megaselia scalaris* (Loew, 1866) | *Rhodnius prolixus* Stal, 1859 | Parasitoidism | Cazorla-Perfetti, Morales-Moreno & Bermúdez-Castillero, 2012 |
| *Megaselia scalaris* (Loew, 1866) | *Triatoma brasiliensis* Neiva, 1911 | Parasitoidism | Costa et al., 2007 |
| *Megaselia* sp. | *Anterhynchium flavomarginatum* (Smith, 1852) | Kleptoparasitism | Itino, 1986 |
| *Megaselia* sp. | *Misumena vatia* (Clerck, 1757) | Parasitoidism | Morse, 2013 |
| *Megaselia* sp. | *Neocorynura tica* Smith-Pardo, 2006 | Kleptoparasitism | Brosi, Smith-Pardo & Gonzalez, 2006 |
| *Megaselia* spp. | *Atta cephalotes* (Linnaeus, 1758) | Parasitoidism | Uribe et al., 2016 |
| *Megaselia steptoeae* Hartop, Brown & Disney, 2015 | *Oxychilus draparnaudi* (Beck, 1837) | Predation | Brown & Vendetti, 2020 |
| *Melaloncha* sp. | *Euglossa cordata* (Linnaeus, 1758) | Parasitoidism | Ament et al., 2014 |
| *Melaloncha* sp. | *Polybia scutellaris* (White, 1841) | Parasitoidism | Lutz & Brown, 2013 |
| *Melaloncha* sp. | *Euglossa townsendi* Cockerell, 1904 | Parasitoidism | Ament et al., 2014 |
| *Melaloncha* sp. | *Centris analis* (Fabricius, 1804) | Parasitoidism | Ament et al., 2014 |
| *Mesopathusa modesta* Disney, 1989 | *Odontotermes transvaalensis* (Sjöstedt, 1902) | Parasitism | Gumming, 1996 |
| *Misotermes mindeni* Disney & Neoh, 2009 | *Macrotermes gilvus* (Hagen, 1858) | Parasitoidism | Foo, Othman & Lee, 2015 |
| *Myriophora communis* Hash & Brown, 2015 | *Narceus americanus* (Palisot de Beauvouis, 1817) | Parasitoidism | Hash et al., 2017 |
| *Myriophora harwoodi* Hash & Brown, 2015 | *Leptogoniulus sorornus* (Butler, 1876) | Parasitoidism | Hash et al., 2017 |
| *Myriophora* sp. | *Atta cephalotes* (Linnaeus, 1758) | Parasitoidism | Uribe et al., 2016 |
| *Myriophora* sp. | *Leptogoniulus sorornus* (Butler, 1876) | Parasitoidism | Hash et al., 2017 |
| *Myrmosicarius brandaoi* Disney, 2006 | *Acromyrmex crassispinus* (Forel, 1909) | Parasitoidism | Elizalde & Folgarait, 2011, 2012 |
| *Myrmosicarius brandaoi* Disney, 2006 | *Acromyrmex fracticornis* (Forel, 1909) | Parasitoidism | Elizalde & Folgarait, 2011, 2012 |
| *Myrmosicarius brandaoi* Disney, 2006 | *Acromyrmex heyeri* (Forel, 1899) | Parasitoidism | Elizalde & Folgarait, 2011, 2012 |
| *Myrmosicarius brandaoi* Disney, 2006 | *Acromyrmex hispidus* Santschi, 1925 | Parasitoidism | Elizalde & Folgarait, 2011, 2012 |
| *Myrmosicarius brandaoi* Disney, 2006 | *Acromyrmex lobicornis* (Emery, 1888) | Parasitoidism | Elizalde & Folgarait, 2011, 2012 |
| *Myrmosicarius brandaoi* Disney, 2006 | *Acromyrmex lundii* (Guérin-Méneville, 1838) | Parasitoidism | Elizalde & Folgarait, 2011, 2012 |
| *Myrmosicarius brandaoi* Disney, 2006 | *Atta vollenweideri* Forel, 1893 | Parasitoidism | Elizalde & Folgarait, 2011, 2012; Guillade & Folgarait, 2011 |
| *Myrmosicarius brandaoi* Disney, 2006 | *Acromyrmex striatus* (Roger, 1863) | Parasitoidism | Elizalde & Folgarait, 2011, 2012 |
| *Myrmosicarius catharinensis* Borgmeier, 1928 | *Acromyrmex ambiguus* (Emery, 1888) | Parasitoidism | Guillade et al., 2017; Goffre & Folgarait, 2019 |
| *Myrmosicarius catharinensis* Borgmeier, 1928 | *Acromyrmex coronatus* (Fabricius, 1804) | Parasitoidism | Elizalde & Queiroz, 2013; Almeida et al., 2021 |
| *Myrmosicarius catharinensis* Borgmeier, 1928 | *Acromyrmex disciger* (Mayr, 1887) | Parasitoidism | Elizalde & Queiroz, 2013 |
| *Myrmosicarius catharinensis* Borgmeier, 1928 | *Acromyrmex lobicornis* (Emery, 1888) | Parasitoidism | Elizalde & Folgarait, 2011, 2012; Guillade et al., 2017 |
| *Myrmosicarius catharinensis* Borgmeier, 1928 | *Acromyrmex lundii* (Guérin-Méneville, 1838) | Parasitoidism | Elizalde & Folgarait, 2011, 2012; Guillade et al., 2017; Goffre & Folgarait, 2019 |
| *Myrmosicarius catharinensis* Borgmeier, 1928 | *Acromyrmex striatus* (Roger, 1863) | Parasitoidism | Elizalde & Folgarait, 2011, 2012 |
| *Myrmosicarius catharinensis* Borgmeier, 1928 | *Atta vollenweideri* Forel, 1893 | Parasitoidism | Elizalde & Folgarait, 2011, 2012 |
| *Myrmosicarius catharinensis* Borgmeier, 1928 | *Acromyrmex fracticornis* (Forel, 1909) | Parasitoidism | Elizalde & Folgarait, 2011, 2012 |
| *Myrmosicarius catharinensis* Borgmeier, 1928 | *Acromyrmex heyeri* (Forel, 1899) | Parasitoidism | Elizalde & Folgarait, 2011, 2012; Guillade et al., 2017; Goffre & Folgarait, 2019 |
| *Myrmosicarius catharinensis* Borgmeier, 1928 | *Acromyrmex crassispinus* (Forel, 1909) | Parasitoidism | Elizalde & Folgarait, 2011, 2012; Guillade et al., 2017; Almeida et al., 2021 |
| *Myrmosicarius catharinensis* Borgmeier, 1928 | *Acromyrmex hispidus* Santschi, 1925 | Parasitoidism | Elizalde & Folgarait, 2011, 2012 |
| *Myrmosicarius catharinensis* Borgmeier, 1928 | Acromyrmex sp. Mayr, 1865 | Parasitoidism | Elizalde et al., 2018 |
| *Myrmosicarius catharinensis* Borgmeier, 1928 | Atta sp. Fabricius, 1804 | Parasitoidism | Elizalde et al., 2018 |
| *Myrmosicarius cristobalensis* Disney, 2006 | *Acromyrmex ambiguus* (Emery, 1888) | Parasitoidism | Guillade et al., 2017; Goffre & Folgarait, 2019 |
| *Myrmosicarius cristobalensis* Disney, 2006 | *Acromyrmex crassispinus* (Forel, 1909) | Parasitoidism | Elizalde & Folgarait, 2011, 2012; Guillade et al., 2017 |
| *Myrmosicarius cristobalensis* Disney, 2006 | *Acromyrmex fracticornis* (Forel, 1909) | Parasitoidism | Elizalde & Folgarait, 2011, 2012 |
| *Myrmosicarius cristobalensis* Disney, 2006 | *Acromyrmex heyeri* (Forel, 1899) | Parasitoidism | Elizalde & Folgarait, 2012; Guillade et al., 2017; Goffre & Folgarait, 2019 |
| *Myrmosicarius cristobalensis* Disney, 2006 | *Acromyrmex hispidus* Santschi, 1925 | Parasitoidism | Elizalde & Folgarait, 2011, 2012 |
| *Myrmosicarius cristobalensis* Disney, 2006 | *Acromyrmex lundii* (Guérin-Méneville, 1838) | Parasitoidism | Elizalde & Folgarait, 2011, 2012; Guillade et al., 2017; Goffre & Folgarait, 2019 |
| *Myrmosicarius cristobalensis* Disney, 2006 | Acromyrmex sp. Mayr, 1865 | Parasitoidism | Elizalde et al., 2018 |
| *Myrmosicarius cristobalensis* Disney, 2006 | *Acromyrmex striatus* (Roger, 1863) | Parasitoidism | Elizalde & Folgarait, 2011, 2012 |
| *Myrmosicarius cristobalensis* Disney, 2006 | Atta sp. Fabricius, 1804 | Parasitoidism | Elizalde et al., 2018 |
| *Myrmosicarius cristobalensis* Disney, 2006 | *Atta vollenweideri* Forel, 1893 | Parasitoidism | Elizalde & Folgarait, 2011, 2012 |
| *Myrmosicarius cristobalensis* Disney, 2006 | *Acromyrmex lobicornis* (Emery, 1888) | Parasitoidism | Elizalde & Folgarait, 2011, 2012; Guillade et al., 2017 |
| *Myrmosicarius crudelis* Borgmeier, 1928 | *Acromyrmex ambiguus* (Emery, 1888) | Parasitoidism | Guillade et al., 2017; Goffre & Folgarait, 2019 |
| *Myrmosicarius crudelis* Borgmeier, 1928 | *Acromyrmex crassispinus* (Forel, 1909) | Parasitoidism | Elizalde & Folgarait, 2011, 2012; Guillade et al., 2017 |
| *Myrmosicarius crudelis* Borgmeier, 1928 | *Acromyrmex fracticornis* (Forel, 1909) | Parasitoidism | Elizalde & Folgarait, 2011, 2012 |
| *Myrmosicarius crudelis* Borgmeier, 1928 | *Acromyrmex heyeri* (Forel, 1899) | Parasitoidism | Elizalde & Folgarait, 2011, 2012; Guillade et al., 2017; Goffre & Folgarait, 2019 |
| *Myrmosicarius crudelis* Borgmeier, 1928 | *Acromyrmex hispidus* Santschi, 1925 | Parasitoidism | Elizalde & Folgarait, 2011, 2012 |
| *Myrmosicarius crudelis* Borgmeier, 1928 | *Acromyrmex lobicornis* (Emery, 1888) | Parasitoidism | Elizalde & Folgarait, 2011, 2012 |
| *Myrmosicarius crudelis* Borgmeier, 1928 | *Acromyrmex lundii* (Guérin-Méneville, 1838) | Parasitoidism | Elizalde & Folgarait, 2011, 2012; Guillade et al., 2017; Goffre & Folgarait, 2019 |
| *Myrmosicarius crudelis* Borgmeier, 1928 | Acromyrmex sp. Mayr, 1865 | Parasitoidism | Elizalde et al., 2018 |
| *Myrmosicarius crudelis* Borgmeier, 1928 | *Acromyrmex striatus* (Roger, 1863) | Parasitoidism | Elizalde & Folgarait, 2011, 2012 |
| *Myrmosicarius crudelis* Borgmeier, 1928 | Atta sp. Fabricius, 1804 | Parasitoidism | Elizalde et al., 2018 |
| *Myrmosicarius crudelis* Borgmeier, 1928 | *Atta vollenweideri* Forel, 1893 | Parasitoidism | Elizalde & Folgarait, 2011, 2012 |
| *Myrmosicarius exrobusta* Brown, 2012 | *Atta robusta* Borgmeier, 1939 | Parasitoidism | Brown, 2012 |
| *Myrmosicarius gonzalezae* Disney, 2006 | *Acromyrmex striatus* (Roger, 1863) | Parasitoidism | Elizalde & Folgarait, 2011, 2012 |
| *Myrmosicarius gonzalezae* Disney, 2006 | *Acromyrmex crassispinus* (Forel, 1909) | Parasitoidism | Elizalde & Folgarait, 2011, 2012 |
| *Myrmosicarius gonzalezae* Disney, 2006 | *Acromyrmex fracticornis* (Forel, 1909) | Parasitoidism | Elizalde & Folgarait, 2011, 2012 |
| *Myrmosicarius gonzalezae* Disney, 2006 | *Acromyrmex heyeri* (Forel, 1899) | Parasitoidism | Elizalde & Folgarait, 2011, 2012 |
| *Myrmosicarius gonzalezae* Disney, 2006 | *Acromyrmex hispidus* Santschi, 1925 | Parasitoidism | Elizalde & Folgarait, 2011, 2012 |
| *Myrmosicarius gonzalezae* Disney, 2006 | *Acromyrmex lobicornis* (Emery, 1888) | Parasitoidism | Elizalde & Folgarait, 2011, 2012 |
| *Myrmosicarius gonzalezae* Disney, 2006 | *Acromyrmex lundii* (Guérin-Méneville, 1838) | Parasitoidism | Elizalde & Folgarait, 2011, 2012 |
| *Myrmosicarius gonzalezae* Disney, 2006 | *Atta vollenweideri* Forel, 1893 | Parasitoidism | Elizalde & Folgarait, 2011, 2012; Guillade & Folgarait, 2011 |
| *Myrmosicarius gracilipes* Borgmeier, 1928 | *Acromyrmex ambiguus* (Emery, 1888) | Parasitoidism | Guillade et al., 2017; Goffre & Folgarait, 2019 |
| *Myrmosicarius gracilipes* Borgmeier, 1928 | *Acromyrmex crassispinus* (Forel, 1909) | Parasitoidism | Guillade et al., 2017 |
| *Myrmosicarius gracilipes* Borgmeier, 1928 | *Acromyrmex heyeri* (Forel, 1899) | Parasitoidism | Guillade et al., 2017; Goffre & Folgarait, 2019 |
| *Myrmosicarius gracilipes* Borgmeier, 1928 | *Acromyrmex lundii* (Guérin-Méneville, 1838) | Parasitoidism | Guillade et al., 2017; Goffre & Folgarait, 2019 |
| *Myrmosicarius grandicornis* Borgmeier, 1928 | *Atta bisphaerica* Forel, 1908 | Parasitoidism | Bragança et al., 2003, 2021; Farder-Gomes et al., 2018 |
| *Myrmosicarius grandicornis* Borgmeier, 1928 | *Atta sexdens* (Linnaeus, 1758) | Parasitoidism | Tonhasca, Bragança & Erthal, 2001; Bragança et al., 2016; Arruda et al., 2019; Galvão et al., 2019; Pereira et al., 2022 |
| *Myrmosicarius grandicornis* Borgmeier, 1928 | *Atta laevigata* (Smith, 1858) | Parasitoidism | Bragança et al., 2016; Arruda et al., 2019; Souza et al., 2021b; Pimentel et al., 2022; de Almeida et al., 2023 |
| *Myrmosicarius longipalpis* Disney, 2006 | *Acromyrmex crassispinus* (Forel, 1909) | Parasitoidism | Elizalde & Folgarait, 2011, 2012 |
| *Myrmosicarius longipalpis* Disney, 2006 | *Acromyrmex fracticornis* (Forel, 1909) | Parasitoidism | Elizalde & Folgarait, 2011, 2012 |
| *Myrmosicarius longipalpis* Disney, 2006 | *Acromyrmex heyeri* (Forel, 1899) | Parasitoidism | Elizalde & Folgarait, 2011, 2012 |
| *Myrmosicarius longipalpis* Disney, 2006 | *Acromyrmex hispidus* Santschi, 1925 | Parasitoidism | Elizalde & Folgarait, 2011, 2012 |
| *Myrmosicarius longipalpis* Disney, 2006 | *Acromyrmex lobicornis* (Emery, 1888) | Parasitoidism | Elizalde & Folgarait, 2011, 2012 |
| *Myrmosicarius longipalpis* Disney, 2006 | *Acromyrmex lundii* (Guérin-Méneville, 1838) | Parasitoidism | Elizalde & Folgarait, 2012; Bragança et al., 2016; Arruda et al., 2019; Souza et al., 2021b; Pimentel et al., 2022; de Almeida et al., 2023 |
| *Myrmosicarius longipalpis* Disney, 2006 | *Acromyrmex striatus* (Roger, 1863) | Parasitoidism | Elizalde & Folgarait, 2011, 2012 |
| *Myrmosicarius longipalpis* Disney, 2006 | *Atta vollenweideri* Forel, 1893 | Parasitoidism | Elizalde & Folgarait, 2011, 2012 |
| *Myrmosicarius persecutor* Borgmeier, 1931 | *Acromyrmex niger* (Smith, 1858) | Parasitoidism | Elizalde & Queiroz, 2013 |
| *Myrmosicarius simplex* Borgmeier & Prado, 1975 | *Acromyrmex niger* (Smith, 1858) | Parasitoidism | Elizalde & Queiroz, 2013 |
| *Myrmosicarius* spp. | *Acromyrmex niger* (Smith, 1858) | Parasitoidism | Barrera et al., 2017 |
| *Myrmosicarius* spp. | *Atta laevigata* (Smith, 1858) | Parasitoidism | Souza & Pereira, 2020 |
| *Myrmosicarius* spp. | *Atta sexdens* (Linnaeus, 1758) | Parasitoidism | Souza & Pereira, 2020 |
| *Myrmosicarius tarsipennis* Borgmeier, 1928 | *Acromyrmex niger* (Smith, 1858) | Parasitoidism | Elizalde & Queiroz, 2013 |
| *Neodorhniphora acromyrmecis* Borgmeier, 1925 | *Acromyrmex niger* (Smith, 1858) | Parasitoidism | Elizalde & Queiroz, 2013 |
| *Neodorhniphora bragancai* Brown, 2001 | *Atta bisphaerica* Forel, 1908 | Parasitoidism | Bragança et al., 2003 |
| *Neodorhniphora curvinervis* Borgmeier, 1969 | *Atta cephalotes* (Linnaeus, 1758) | Parasitoidism | Orr, 1992; Feener & Brown, 1993 |
| *Neodorhniphora declinata* Borgmeier, 1925 | *Atta laevigata* (Smith, 1858) | Parasitoidism | Silva et al., 2008 |
| *Neodorhniphora elongata* Brown, 2001 | *Atta sexdens rubropilosa* Forel, 1908 | Parasitoidism | Silva et al., 2008; Bragança, Tonhasca & Lucia, 2009b; Gazal, Bailez & Viana-Bailez, 2009 |
| *Neodorhniphora erthali* Brown, 2001 | *Atta laevigata* (Smith, 1858) | Parasitoidism | Braganca, Tonhasca Jr & Moreira, 2002 |
| *Neodorhniphora erthali* Brown, 2001 | *Atta sexdens* (Linnaeus, 1758) | Parasitoidism | Vieira-Neto, Mundim & Vasconcelos, 2006 |
| *Neodorhniphora similis* Prado, 1976 | *Acromyrmex niger* (Smith, 1858) | Parasitoidism | Elizalde & Queiroz, 2013 |
| *Neodorhniphora* spp. | *Acromyrmex niger* (Smith, 1858) | Parasitoidism | Barrera et al., 2017 |
| *Neodorhniphora* spp. | *Atta sexdens* (Linnaeus, 1758) | Parasitoidism | Braganca, Tonhasca & Della Lucia, 1998; Tonhasca Jr & Bragança, 2000 |
| *Neodorhniphora* spp. | *Atta sexdens rubropilosa* Forel, 1908 | Parasitoidism | Silva et al., 2007; Braganca et al., 2008 |
| *Neodorhniphora tonhascai* Brown, 2001 | *Atta laevigata* (Smith, 1858) | Parasitoidism | Braganca et al., 2002; Silva et al., 2008 |
| *Neodorhniphora tonhascai* Brown, 2001 | *Acromyrmex crassispinus* (Forel, 1909) | Parasitoidism | Silva et al., 2008 |
| *Neodorhniphora tonhascai* Brown, 2001 | *Acromyrmex subterraneus molestans* Santschi, 1928 | Parasitoidism | Silva et al., 2008 |
| *Neodorhniphora unichaeta* Disney, 2009 | *Acromyrmex ambiguus* (Emery, 1888) | Parasitoidism | Guillade et al., 2017 |
| *Neodorhniphora unichaeta* Disney, 2009 | *Acromyrmex crassispinus* (Forel, 1909) | Parasitoidism | Elizalde & Folgarait, 2011; Guillade et al., 2017 |
| *Neodorhniphora unichaeta* Disney, 2009 | *Acromyrmex fracticornis* (Forel, 1909) | Parasitoidism | Elizalde & Folgarait, 2011 |
| *Neodorhniphora unichaeta* Disney, 2009 | *Acromyrmex heyeri* (Forel, 1899) | Parasitoidism | Elizalde & Folgarait, 2011; Guillade et al., 2017 |
| *Neodorhniphora unichaeta* Disney, 2009 | *Acromyrmex hispidus* Santschi, 1925 | Parasitoidism | Elizalde & Folgarait, 2011 |
| *Neodorhniphora unichaeta* Disney, 2009 | *Acromyrmex lobicornis* (Emery, 1888) | Parasitoidism | Elizalde & Folgarait, 2011 |
| *Neodorhniphora unichaeta* Disney, 2009 | *Acromyrmex lundii* (Guérin-Méneville, 1838) | Parasitoidism | Elizalde & Folgarait, 2011; Guillade et al., 2017 |
| *Neodorhniphora unichaeta* Disney, 2009 | Acromyrmex sp. Mayr, 1865 | Parasitoidism | Elizalde et al., 2018 |
| *Neodorhniphora unichaeta* Disney, 2009 | *Acromyrmex striatus* (Roger, 1863) | Parasitoidism | Elizalde & Folgarait, 2011 |
| *Neodorhniphora unichaeta* Disney, 2009 | Atta sp. Fabricius, 1804 | Parasitoidism | Elizalde et al., 2018 |
| *Neodorhniphora unichaeta* Disney, 2009 | *Atta vollenweideri* Forel, 1893 | Parasitoidism | Elizalde & Folgarait, 2011 |
| *Neopleurophora* sp. | *Atta cephalotes* (Linnaeus, 1758) | Parasitoidism | Uribe et al., 2016 |
| *Phalacrotophora epeirae* (Brues, 1902) | *Mimetus notius* Chamberlin, 1923 | Predation | Guarisco, 2001 |
| *Phalacrotophora fasciata* (Fallén, 1823) | *Harmonia axyridis* (Pallas, 1773) | Parasitoidism | Francati, 2015 |
| *Phalacrotophora halictorum* (Melander & Brues, 1903) | *Lasioglossum figueresi* Wcislo, 1990 | Kleptoparasitism | Wcislo, 1990 |
| *Pseudacteon affinis* Borgmeier, 1962 | *Solenopsis saevissima* (Smith, 1855) | Parasitoidism | Pesquero, Vaz & De Arruda, 2013 |
| *Pseudacteon bifidus* Brown and Morrison, 1999 | *Solenopsis geminata* (Fabricius, 1804) | Parasitoidism | Morrison, 1999 |
| *Pseudacteon borgmeieri* Schmitz, 1923 | *Solenopsis invicta* Buren, 1972 | Parasitoidism | Morrison & Gilbert, 1999; Wuellner, Porter & Gilbert, 2002 |
| *Pseudacteon borgmeieri* Schmitz, 1923 | *Solenopsis richteri* Forel, 1909 | Parasitoidism | Folgarait & Gilbert, 1999; Folgarait, Bruzzone & Gilbert, 2003 |
| *Pseudacteon borgmeieri* Schmitz, 1923 | *Solenopsis saevissima* (Smith, 1855) | Parasitoidism | Orr, Seike & Gilbert, 1997 |
| *Pseudacteon browni* Disney, 1991 | *Solenopsis geminata* (Fabricius, 1804) | Parasitoidism | Morrison & Gilbert, 1998, 1999; Morrison, 1999 |
| *Pseudacteon calderensis* Calcaterra, 2007 | *Solenopsis interrupta* Santschi, 1916 | Parasitoidism | Calcaterra, 2007 |
| *Pseudacteon comatus* Borgmeier, 1925 | *Solenopsis richteri* Forel, 1909 | Parasitoidism | Folgarait & Gilbert, 1999; Folgarait et al., 2003 |
| *Pseudacteon crawfordi* Coquillett, 1907 | *Solenopsis geminata* (Fabricius, 1804) | Parasitoidism | Morrison, 1999 |
| *Pseudacteon cultellatus* Borgmeier, 1925 | *Solenopsis invicta* Buren, 1972 | Parasitoidism | Folgarait, Bruzzone & Gilbert, 2002a |
| *Pseudacteon cultellatus* Borgmeier, 1925 | *Solenopsis saevissima* (Smith, 1855) | Parasitoidism | Pesquero, Vaz & De Arruda, 2013 |
| *Pseudacteon cultellatus* Borgmeier, 1925 | Solenopsis spp. Westwood, 1840 | Parasitoidism | Ngumbi & Fadamiro, 2015 |
| *Pseudacteon cultellatus* Borgmeier, 1926 | *Solenopsis richteri* Forel, 1909 | Parasitoidism | Folgarait et al., 2002a |
| *Pseudacteon curvatus* Borgmeier, 1925 | S. richteri × S. invicta (Hybrid) | Parasitoidism | Thead & Streett, 2005; He & Fadamiro, 2009; Chen et al., 2012 |
| *Pseudacteon curvatus* Borgmeier, 1925 | *Solenopsis geminata* (Fabricius, 1804) | Parasitoidism | Ajayi, Chen & Fadamiro, 2020 |
| *Pseudacteon curvatus* Borgmeier, 1925 | *Solenopsis invicta* Buren, 1972 | Parasitoidism | Wuellner et al., 2002; Vogt et al., 2003; Chirino, Gilbert & Folgarait, 2009; He & Fadamiro, 2009; LeBrun, Plowes & Gilbert, 2009; Puckett & Harris, 2010; Valles, Oi & Porter, 2010; Plowes, LeBrun & Gilbert, 2011; Chen et al., 2012; Puckett, Harris & Gold, 2012; Sharma & Fadamiro, 2013; Oi et al., 2019; Ajayi et al., 2020 |
| *Pseudacteon curvatus* Borgmeier, 1925 | *Solenopsis richteri* Forel, 1909 | Parasitoidism | Folgarait & Gilbert, 1999; Folgarait et al., 2003; Vogt & Streett, 2003; He & Fadamiro, 2009; Chen et al., 2012; Ajayi et al., 2020 |
| *Pseudacteon curvatus* Borgmeier, 1925 | *Solenopsis saevissima* (Smith, 1855) | Parasitoidism | Fowler, 1997 |
| *Pseudacteon curvatus* Borgmeier, 1925 | Solenopsis spp. Westwood, 1840 | Parasitoidism | Ngumbi & Fadamiro, 2015 |
| *Pseudacteon dentiger* Borgmeier, 1962 | *Solenopsis saevissima* (Smith, 1855) | Parasitoidism | Pesquero, Vaz & De Arruda, 2013 |
| *Pseudacteon disneyi* Pesquero, 2000 | *Solenopsis saevissima* (Smith, 1855) | Parasitoidism | Pesquero, Vaz & De Arruda, 2013 |
| *Pseudacteon formicarum* (Verrall, 1877) | *Lasius emarginatus* (Olivier, 1792) | Parasitoidism | Weissflog et al., 2008 |
| *Pseudacteon fowleri* Pesquero, 2000 | *Solenopsis saevissima* (Smith, 1855) | Parasitoidism | Pesquero, Vaz & De Arruda, 2013 |
| *Pseudacteon lasciniosus* (Brown & Philpott, 2012) | *Azteca sericeasur* Longino, 2007 | Parasitoidism | Mathis & Tsutsui, 2016 |
| *Pseudacteon lenkoi* Borgmeier & Prado, 1975 | *Solenopsis saevissima* (Smith, 1855) | Parasitoidism | Pesquero, Vaz & De Arruda, 2013 |
| *Pseudacteon litoralis* Borgmeier, 1925 | *Solenopsis invicta* Buren, 1972 | Parasitoidism | Morrison & Gilbert, 1998; Wuellner et al., 2002 |
| *Pseudacteon litoralis* Borgmeier, 1925 | *Solenopsis richteri* Forel, 1909 | Parasitoidism | Folgarait & Gilbert, 1999 |
| *Pseudacteon litoralis* Borgmeier, 1925 | *Solenopsis saevissima* (Smith, 1855) | Parasitoidism | Pesquero et al., 1995; Porter et al., 1995; Fowler, 1997; Orr et al., 1997 |
| *Pseudacteon lontrae* Mattos & Orr, 2002 | *Linepithema piliferum* (Mayr, 1870) | Parasitoidism | Orr, Dahlsten & Benson, 2003 |
| *Pseudacteon nocens* Borgmeier, 1926 | *Solenopsis invicta* Buren, 1972 | Parasitoidism | Plowes, Folgarait & Gilbert, 2012 |
| *Pseudacteon nudicornis* Borgmeier, 1925 | *Solenopsis invicta* Buren, 1972 | Parasitoidism | Wuellner et al., 2002 |
| *Pseudacteon nudicornis* Borgmeier, 1925 | *Solenopsis richteri* Forel, 1909 | Parasitoidism | Folgarait & Gilbert, 1999; Folgarait et al., 2003 |
| *Pseudacteon nudicornis* Borgmeier, 1925 | *Solenopsis saevissima* (Smith, 1855) | Parasitoidism | Orr et al., 1997; Pesquero et al., 2013 |
| *Pseudacteon obtusus* Borgmeier, 1925 | *Solenopsis geminata* (Fabricius, 1804) | Parasitoidism | Morrison & Gilbert, 1999; Ajayi et al., 2020 |
| *Pseudacteon obtusus* Borgmeier, 1925 | *Solenopsis invicta* Buren, 1972 | Parasitoidism | Morrison & Gilbert, 1999; Wuellner et al., 2002; Folgarait et al., 2005; Valles et al., 2010; Plowes et al., 2011; Sharma & Fadamiro, 2013; Oi et al., 2019; Ajayi et al., 2020 |
| *Pseudacteon obtusus* Borgmeier, 1925 | *Solenopsis richteri* Forel, 1909 | Parasitoidism | Folgarait et al., 2003, 2005; Ajayi et al., 2020 |
| *Pseudacteon obtusus* Borgmeier, 1925 | *Solenopsis saevissima* (Smith, 1855) | Parasitoidism | Porter et al., 1995; Orr et al., 1997 |
| *Pseudacteon obtusus* Borgmeier, 1925 | Solenopsis spp. Westwood, 1840 | Parasitoidism | Ngumbi & Fadamiro, 2015 |
| *Pseudacteon pradei* Borgmeier, 1925 | *Solenopsis saevissima* (Smith, 1855) | Parasitoidism | Fowler, 1997 |
| *Pseudacteon pusillus* Borgmeier, 1938 | Linepithema sp. Mayr, 1866 | Parasitoidism | Orr et al., 2003 |
| *Pseudacteon solenopsidis* (Schmitz, 1914) | *Solenopsis invicta* Buren, 1972 | Parasitoidism | Wuellner et al., 2002 |
| *Pseudacteon solenopsidis* (Schmitz, 1914) | *Solenopsis saevissima* (Smith, 1855) | Parasitoidism | Orr et al., 1997 |
| *Pseudacteon* sp. | *Atta cephalotes* (Linnaeus, 1758) | Parasitoidism | Uribe et al., 2016 |
| *Pseudacteon* sp. | *Azteca instabilis* (Smith, 1862) | Parasitoidism | Mathis, Philpott & Moreira, 2011 |
| *Pseudacteon spatulatus* (Malloch, 1912) | *Solenopsis geminata* (Fabricius, 1804) | Parasitoidism | Morrison, 1999 |
| *Pseudacteon* spp. | *Azteca instabilis* (Smith, 1862) | Parasitoidism | Philpott et al., 2004; Philpott, Pardee & Gonthier, 2012; Philpott, 2005; Mathis et al., 2011 |
| *Pseudacteon* spp. | *Linepithema humile* (Mayr, 1868) | Parasitoidism | Orr et al., 2001 |
| *Pseudacteon* spp. | *Solenopsis geminata* (Fabricius, 1804) | Parasitoidism | Morrison & Gilbert, 1998; Morrison et al., 1999, 2000 |
| *Pseudacteon tricuspis* Borgmeier, 1925 | *Forelius mccooki* (McCook, 1879) | Parasitoidism | Mehdiabadi, Kawazoe & Gilbert, 2004 |
| *Pseudacteon tricuspis* Borgmeier, 1925 | S. richteri × S. invicta (Hybrid) | Parasitoidism | He & Fadamiro, 2009; Chen et al., 2012 |
| *Pseudacteon tricuspis* Borgmeier, 1925 | *Solenopsis invicta* Buren, 1972 | Parasitoidism | Porter, Williams & Patterson, 1997; Morrison & Gilbert, 1998; Morrison, 2000; Cônsoli et al., 2001; Wuellner et al., 2002a; Mehdiabadi & Gilbert, 2002; Smith & Gilbert, 2003; Vogt et al., 2003; Morrison & King, 2004; Mottern, Heinz & Ode, 2004; Barr & Calixto, 2005; Morrison & Porter, 2005, 2006; Ramirez, Thompson & Remmenga, 2006; Chen & Fadamiro, 2007; Chen, Sharma & Fadamiro, 2009; Chen et al., 2012; He & Fadamiro, 2009; Henne & Johnson, 2009; LeBrun et al., 2009; Puckett & Harris, 2010; Valles et al., 2010; Plowes et al., 2011; Sharma, Vander Meer & Fadamiro, 2011; Puckett et al., 2012; Sharma & Fadamiro, 2013 |
| *Pseudacteon tricuspis* Borgmeier, 1925 | *Solenopsis richteri* Forel, 1909 | Parasitoidism | Porter et al., 1997; Folgarait & Gilbert, 1999; Folgarait et al., 2003; He & Fadamiro, 2009; Chen et al., 2012 |
| *Pseudacteon tricuspis* Borgmeier, 1925 | *Solenopsis saevissima* (Smith, 1855) | Parasitoidism | Porter et al., 1995; Fowler, 1997; Orr et al., 1997; Pesquero et al., 2013 |
| *Pseudacteon tricuspis* Borgmeier, 1925 | Solenopsis spp. Westwood, 1840 | Parasitoidism | Ngumbi & Fadamiro, 2015 |
| *Pseudacteon wasmanni* (Schmitz, 1914) | *Solenopsis invicta* Buren, 1972 | Parasitoidism | Morrison & Gilbert, 1998; Wuellner et al., 2002a |
| *Pseudacteon wasmanni* (Schmitz, 1914) | *Solenopsis saevissima* (Smith, 1855) | Parasitoidism | Pesquero et al., 1995; Porter et al., 1995; Fowler, 1997; Orr et al., 1997 |
| *Pseudohypocera kerteszi* (Enderlein, 1912) | *Melipona fasciculata* Smith, 1854 | Kleptoparasitism | Oliveira, Venturieri & Contrera, 2013 |
| *Pseudohypocera kerteszi* (Enderlein, 1912) | *Melipona seminigra* Friese, 1903 | Kleptoparasitism | Oliveira, Venturieri & Contrera, 2013 |
| *Puliciphora* spp. | *Atta cephalotes* (Linnaeus, 1758) | Parasitoidism | Uribe et al., 2016 |
| *Razorfemora zaragozae* Disney, 2006 | *Messor barbarus* (Linnaeus, 1767) | Parasitoidism | Delgado, Disney & Palma, 2020 |
| *Rhyncophoromyia maculineura* Borgmeier, 1959 | *Camponotus sericeiventris* (Guérin-Méneville, 1838) | Parasitoidism | Brown & Feener Jr, 1993 |
| *Sarcophaga aldrichi* Parker, 1916 | *Cucullia lucifuga* (Denis & Schiffermüller, 1775) | Parasitoidism | Mccabe, 1998 |
| *Synclinusa* sp. | *Atta cephalotes* (Linnaeus, 1758) | Parasitoidism | Uribe et al., 2016 |
| *Termitophilomyia zimbraunsi* Disney, 1994 | *Odontotermes transvaalensis* (Sjöstedt, 1902) | Parasitism | Gumming, 1996 |

**References**
